# The emergence of human primordial germ cell–like cells in stem cell–derived gastruloids

**DOI:** 10.1126/sciadv.ado1350

**Published:** 2025-03-26

**Authors:** Jitesh Neupane, Gabriele Lubatti, Theresa Gross-Thebing, Mayra Luisa Ruiz Tejada Segura, Richard Butler, Sargon Gross-Thebing, Sabine Dietmann, Antonio Scialdone, M. Azim Surani

**Affiliations:** ^1^ Gurdon Institute, University of Cambridge, Cambridge CB2 1QN, UK.; ^2^ Physiology, Development and Neuroscience Department, University of Cambridge, Cambridge, UK.; ^3^ Institute of Epigenetics and Stem Cells, Helmholtz Zentrum München, 81377 Munich, Germany.; ^4^ Institute of Computational Biology, Helmholtz Zentrum München, 85764 Neuherberg, Germany.; ^5^ Institute of Functional Epigenetics, Helmholtz Zentrum München, 85764 Neuherberg, Germany.; ^6^ Department of Development Biology, Washington University School of Medicine in St. Louis, St. Louis, MO, USA.

## Abstract

Most advances in early human postimplantation development depend on animal studies and stem cell–based embryo models. Here, we present self-organized three-dimensional human gastruloids (hGs) derived from embryonic stem cells. The transcriptome profile of day 3 hGs aligned with Carnegie stage 7 human gastrula, with cell types and differentiation trajectories consistent with human gastrulation. Notably, we observed the emergence of nascent primordial germ cell–like cells (PGCLCs), but without exogenous bone morphogenetic protein (BMP) signaling, which is essential for the PGCLC fate. A mutation in the *ISL1* gene affects amnion-like cells and leads to a loss of PGCLCs; the addition of exogenous BMP2 rescues the PGCLC fate, indicating that the amnion may provide endogenous BMP signaling. Our model of early human embryogenesis will enable further exploration of the germ line and other early human lineages.

## Teaser

3D human gastruloids mimic gastrulation and postgastrulation development, forming the three germ layer derivatives and hPGCLCs.

## INTRODUCTION

The human body plan is established following blastocyst implantation on embryonic days 6 and 7 (E6 and E7, respectively). During this period, the embryo develops from a bilaminar epiblast-hypoblast disc into a trilaminar disc that includes three germ layers: ectoderm, mesoderm, and endoderm. At gastrulation, epiblast cells invaginate and undergo epithelial-to-mesenchymal transition, giving rise to primitive streak (PS) and the formation of mesoderm and endoderm. Epiblast cells simultaneously migrate dorsally to form squamous epithelial cells of amniotic ectoderm, eventually creating an amnion cavity (*1*, *2*). Primordial germ cells (PGCs), the precursors of sperm and eggs, also emerge around day 14 (D14) of gastrulation in response to bone morphogenetic protein (BMP) signaling (*3*–*5*). Technical and ethical challenges hinder investigations involving early human embryos. These challenges could be addressed using models that simulate features of perigastrulation development.

Human PGC-like cells (hPGCLCs) have been identified in embryoid bodies generated from pluripotent stem cells (PSCs), in which the presence of exogenous BMP is essential (*6*–*8*). Recent integrated stem cell–based embryo models with extraembryonic tissues that mimic human postimplantation development have reported PGCLCs on D14 (*9*–*11*), although some tissues, such as extraembryonic tissues and PGCLCs, were induced in response to overexpression of transcription factors or expression of transgenes. Similarly, micropatterned and microfluidics-based embryo models (*12*, *13*) for epiblast amniogenesis (*14*, *15*) have also shed light on early development, while a bioengineered culture system reported the induction of hPGCLCs through paracrine signaling downstream of *ISL1* (*16*). On the other hand, a two-dimensional (2D) gastruloid (or micropatterned) model displayed the emergence of hPGCLCs in the presence of exogenous BMP4 (*17*). Notably, a rare live Carnegie stage 8 (CS8) human embryo has shown the presence of hPGCs at the site of the connecting stalk, a structure that connects an embryo to the shell of the trophoblast cells (*18*).

While the Carnegie collection of human embryos offers valuable descriptive information, our understanding of the critical events in early human development remains limited. Recent in vitro models, including blastoids (*19*–*22*), embryoids (*23*–*25*), and gastruloids, effectively mimic human embryonic development both before and after gastrulation. These models can be used to gain insights into the origin of PGCs.

Here, we present self-organized 3D human gastruloids (hGs) derived from human embryonic stem cells (hESCs). These gastruloids mimic gastrulation and postgastrulation development, successfully forming the three germ layers and generating hPGCLCs without exogenous BMP supplementation. This process appears to occur in response to endogenous BMP signaling driven by the amnion. Our study provides a previously unidentified approach for investigating critical aspects of human development during and after gastrulation, including the origins and specification of hPGCLCs.

## RESULTS

### Generation of hGs

**Fig. 1.**
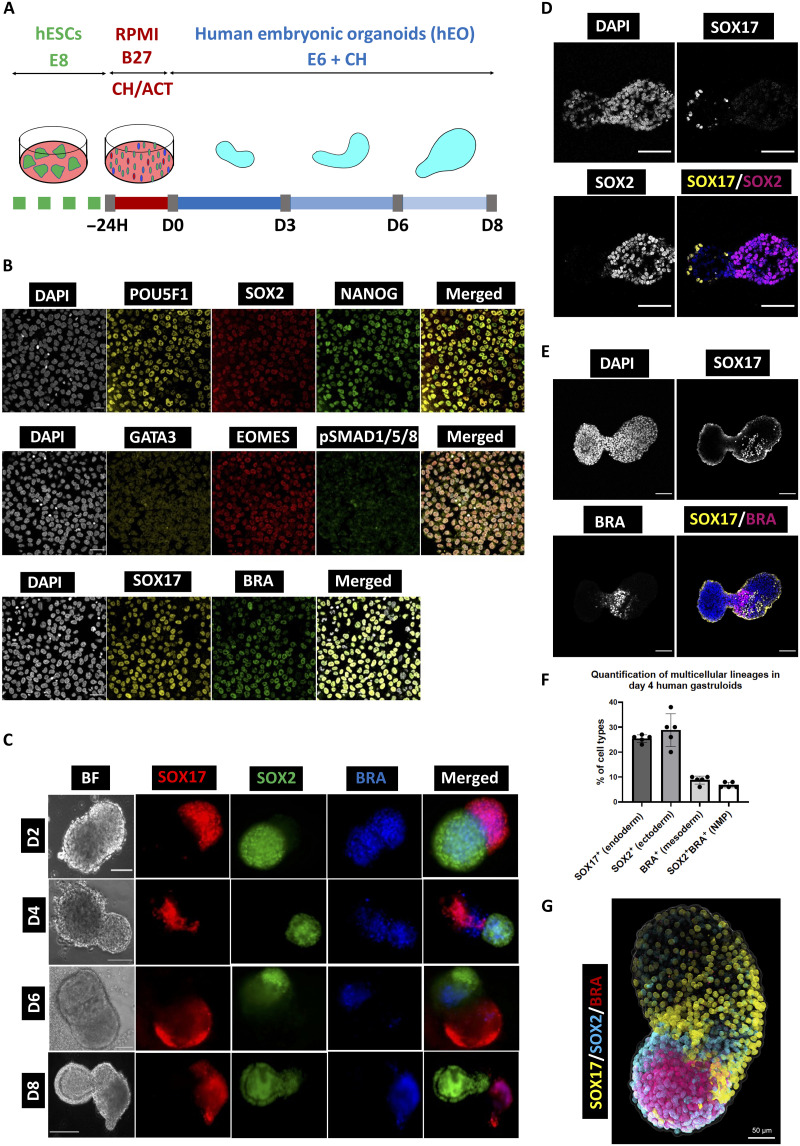
Self-organization of hESCs into 3D gastruloids establishes primary germ layer derivatives. (**A**) Experimental plan showing the generation of the hG model from hESCs in vitro. Ch, Chiron (CHIR99021); Act, Activin A; E8, Essential 8 Medium; RB27, Advanced RPMI Medium supplemented with B27. H, hours. (**B**) IF staining of transiently induced hESCs showing pluripotent markers stained for POU5F1 (OCT4), SOX2, and NANOG (top); mesendoderm markers EOMES, SOX17, and *BRACHYUARY* (*BRA*) (bottom); and GATA3 and pSMAD1/5/8, which are negative (middle). Scale bars, 50 μm (*n* = 15 samples from three experiments). (**C**) Formation of the three germ layer derivatives on hGs from D2 to D8 derived from *RUES2-GLR* hESCs. Reporter colors represent ectoderm (*SOX2-mCitrine*), mesoderm (*BRA-mCerulean*), and endoderm (*SOX17-tdTomato*). Scale bars, 250 μm (D2, *N* = 1620; D4, *N* = 1100, *n* = 10 experiments). (**D** and **E**) Representative confocal micrographs showing the primary germ layer derivatives (endoderm, ectoderm, and mesoderm) at D4 hG sections derived from *W15-tdTomato hESCs* stained for SOX17, SOX2, and BRA (or BRA), respectively. Scale bars, 200 μm (C) and 100 μm (D) (*n* = 10 samples from three experiments). (**F**) Quantification of multicellular lineages detected in D4 hGs [segmented from 39,822 cells (nuclei) from five images]. (**G**) Projection of D4 hGs representing multicellular lineages in (F). Cyan, ectoderm (SOX2^+^); yellow, endoderm (SOX17^+^); magenta, mesoderm (BRA^+^).

We previously demonstrated that transient induction of human PSCs by *Nodal* agonist *ACTIVIN-A* (*ACT*) and *WNT* agonist *CHIRON* (*CH*) up-regulates the expression of the PS markers, including BRACHYURY (BRA or T) and EOMES (*7*). Recent studies have also shown that a *CH* pulse induces PS (*26*) and gastruloids (*27*). We transiently induced mesendodermal genes by culturing hESCs for 24 hours in the presence of *CH* and *ACT* (Fig. 1A) (*28*, *29*). These cells showed heterogeneous expression of BRA, EOMES, and SOX17, together with a reduced expression of pluripotency genes, POU5F1 (OCT4), NANOG, and SOX2 (Fig. 1B and fig. S1A).

Because high *Wnt* activity can prevent hPGCLC induction in favor of the endoderm lineage (*7*, *30*), we assessed the developmental capacity of these structures with a reduced *CH* dosage (*27*). Aggregates did not continue to extend without *CH* (designated as 0.00 μM in fig. S1B), as expected (*27*), but lower dosages of CH (0.25, 0.5, and 1.0 μM) promoted the self-organization of hESCs into 3D elongated structures. The 0.25 μM *CH* was selected for all further experiments.

We used *RUES2* hESC with *SOX2-mCitrine*, *TBXT-mCerulean*, and *SOX17-tdTomato* reporters (*31*) to track the development and progression of the three germ layer derivatives in hGs. In the early stages of differentiation on D2, we observed self-organized 3D structures with expression of SOX2, TBXT, and SOX17, representing the ectoderm, mesoderm, and endoderm, respectively (Fig. 1C and fig. S1, C and D). These structures consisted of distinct groups of cells that occupied different, partially overlapping domains. After 2 days, more than 90% (1620 of 1800, *n* = 10 experiments) of the aggregates elongated along the longitudinal axis (Fig. 1C and fig. S1D). On D4, ~60% of the aggregates displayed all three germ layer derivatives, as indicated by the expression of triple germ layer reporters (*31*). Immunofluorescence (IF) analysis of the hG sections using the *W15-NANOS3-tdTomato* hESC line also confirmed the expression of SOX2, TBXT, and SOX17, establishing three germ layer derivatives (Fig. 1, D and E). We quantified the formation of multicellular lineages (Fig. 1F) on the basis of a 3D projection of D4 hGs (Fig. 1G and movie S1) that displayed ~30% SOX2^+^ ectoderm, ~28% SOX17^+^ endoderm, and ~10% BRA^+^ mesoderm derivatives. BRA and SOX2 were expressed at one end, whereas SOX17 was expressed at the opposite end. We also observed ~8% SOX2^+^BRA^+^ double-positive cells, potentially representing neuromesodermal progenitors (NMPs) at the posterior end of the hG (Fig. 1, F and G).

### Transcriptome analysis at a single-cell resolution

**Fig. 2.**
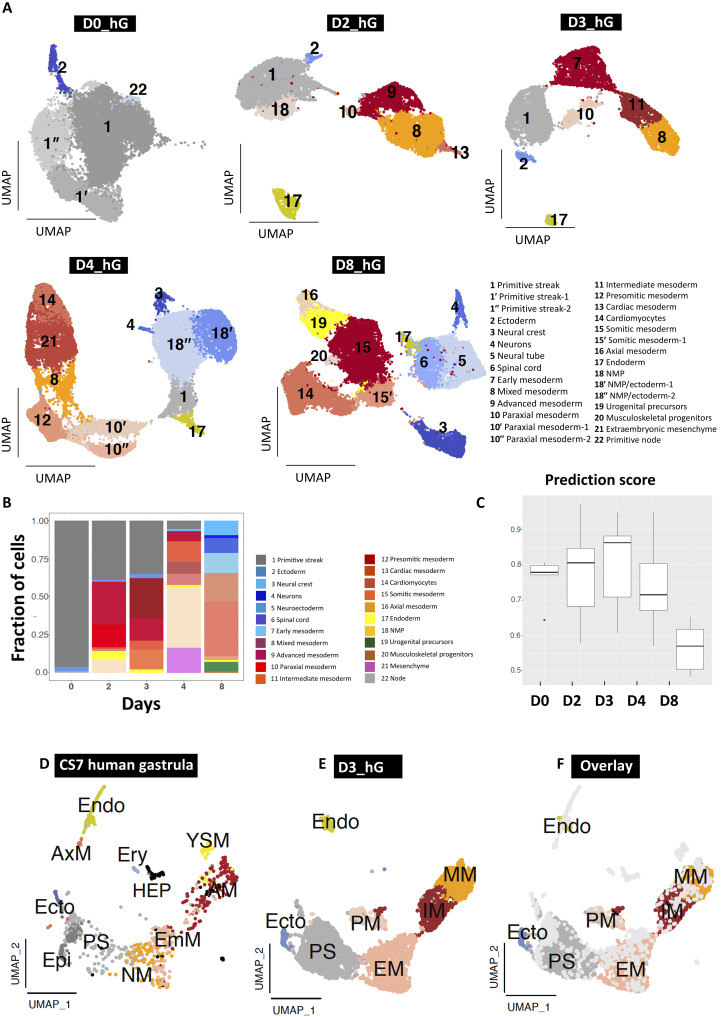
Transcriptional characterization of hGs. (**A**) Uniform manifold approximation and projection (UMAP) plots showing the clustering and cell type annotation of hGs sampled on D0, D2, D3, D4, and D8 obtained by 10× scRNA-seq. (**B**) Proportion of cell types detected in hGs at different time points of in vitro development from D0 to D8. (**C**) Plot showing the prediction score based on the mapping of cell types detected in CS7 human gastrula and hGs at different time points. (**D** to **F**) Coembedding in a UMAP plot of the single-cell transcriptomes from a CS7 human gastrula and D3 hGs. Endo, endoderm; Ecto, ectoderm; EM, early mesoderm; PM, paraxial mesoderm; IM, intermediate mesoderm; MM, mixed mesoderm; AxM, axial mesoderm; YSM, yolk sac mesoderm; EmM, emergent mesoderm; NM, nascent mesoderm; Epi, epiblast; Ery, erythrocytes; HEP, hemogenic endothelial progenitors.

To investigate the organization, dynamics, and cellular complexity of the developing hGs, we performed 10× single-cell RNA sequencing (scRNA-seq), sampled on D0, D2, D3, D4, and D8 (Fig. 2A). After performing quality control (figs. S2 and S3), we identified 22 cell populations that emerged gradually over time (Fig. 2B). D0 represents the transient stage at 24 hours in the presence of ACT and CH (Fig. 1A and fig. S4A), marking the beginning of the exit from pluripotency and the onset of differentiation (Fig. 2, A and B, and fig. S4B). From D2 onward, the self-organization of hGs led to the emergence of different germ layer derivatives, including features of gastrulation and early neurulation (Figs. 1C and 2A).

To validate the development of hGs with the in vivo counterpart, we mapped the transcriptome of hGs onto a CS7 human gastrula (Fig. 2C) (*4*) and computed a score that represents the overall transcriptional similarity (see Materials and Methods). We found that the highest similarity score corresponds to D3 hGs (Fig. 2, C to F). In particular, emergent mesoderm (EmM) and nascent mesoderm (NM) in CS7 gastrula aligned to early mesoderm (EM) in hGs, whereas advanced mesoderm (AM) in CS7 gastrula aligned to intermediate mesoderm (IM) and mixed mesoderm (MM) in hGs. We compared our dataset with previously described hGs (*27*) and CS8 to CS11 nonhuman primate embryos (*32*). Our analysis revealed that D3 hG cell types mapped with PS, early mesoderm, mixed mesoderm, and paraxial mesoderm. On the other hand, D4 hG cell types mapped with paraxial mesoderm and presomitic mesoderm according to previously published hGs’ tomo-seq data (fig. S4C). Furthermore, when we mapped our data onto a dataset from CS11 NHP embryos, we found that the closest cell types to neural crest cells and neuronal cells in D8 hGs are neural crest cells and spinal cord (fig. S4D) (*32*). While we detected amnion-like cells (AMLCs) in the hGs, the model lacked other extraembryonic lineages, such as trophoblast, yolk-sac endoderm, and visceral endoderm cells.

The induced cells on D0 showed expression of ectodermal markers, including *ZIC1* and *EN2* (fig. S4E). Within the PS cluster on D0, markers associated with both anterior and posterior PS, including *SOX17*, *FOXC1*, and *TBX6*, were expressed (fig. S4F). The heterogeneous expression of pluripotency and PS markers (Fig. 2B and fig. S1A) on D0 indicated a transient state following exit from pluripotency and the onset of differentiation (fig. S4G).

Overall, cells in the hGs primarily represented the PS (fig. S5A), mesoderm (fig. S5B), ectoderm (fig. S5, C and D), and endoderm (fig. S5E). Mesodermal cells were further classified into early, intermediate, paraxial, presomitic, and cardiac mesoderm (fig. S5B). Ectodermal cells were subclassified into neural tube, neural crest, and neuronal precursors (fig. S5C).

### Emergence of NMPs in gastruloids

We used the transcriptomic signature of rostral caudal mesoderm from CS7 human gastrula and compared with the intermediate and mixed mesoderm clusters on D3, the hG stage closest to CS7 (see Fig. 2, C to F). Using the Seurat classifier trained on the CS7 human gastrula dataset (see Materials and Methods), most cells in the intermediate mesoderm and ~50% of cells in the mixed mesoderm were classified as “caudal” (fig. S5, F and G). In addition, we noted differences in rostral genes (*GATA6*, *MYH10*, *TNNI1*, *TNNT2*, and *HAND1*) and caudal genes (*CDX2* and *CDX1*) within these clusters (fig. S5H). This indicates that the hGs display a transcriptional signature along the rostro-caudal axis that is similar to that observed in human embryos. To verify the presence of the rostro-caudal axis in hGs, we performed IF studies. We detected the expression of BRA and CDX2 in the posterior region (*27*, *33*) and GATA6 expression in the anterior region (*27*). As in the previous gastruloid model (*27*), we observed GATA6^+^ cells at one pole and cells with BTRA^+^ or CDX2^+^ expression at the opposite pole (fig. S5I), indicating an elongation and differential cell fates along the rostro-caudal axis.

We identified NMPs in the caudal region of hGs by detecting the coexpression of a neural factor SOX2 and a mesodermal factor Brachyury (BRA) (*33*, *34*) by IF (Fig. 1, F and G, and fig. S6A), as reported previously (*33*). In our scRNA-seq dataset, we detected *TBXT^+^SOX2^+^*, *SOX2^+^NKX1-2^+^*, and *TBXT^+^NKX1-2^+^* cells on D2 to D4, confirming the presence of NMPs in the hGs (fig. S6B). We observed the NMP markers (*TBXT*, *SOX2*, and *NKX1-2*) as early as D2 (fig. S6C).

NMPs originate at the anterior PS of mammalian embryos (*35*) and contribute to both neural and mesodermal progenitors (*36*). In CS7 human gastrula (*4*) and previously published hGs (*27*), neuronal precursor expression was not detected (Fig. 2, A, B, and D, and fig. S4D). However, we observed the expression of neural crest markers *S100B* and *SOX10* (fig. S6D), as well as neuronal markers like *ELAVL3* and *NEUROD4* (fig. S6E).

By D8, we detected derivatives of neuroectodermal lineage, including the neural crest markers (*FOXD3* and *SOX10*) (fig. S6F), neuronal precursors (*STMN2* and *NEUROD4)* (fig. S6G), neural ectoderm (*SOX3* and *PAX6*) (fig. S6H), and spinal cord precursors (*OLIG3* and *SOX1*) (fig. S6I). We confirmed the expression of SOX1- and PAX6-positive neural ectoderm and SOX2- and TFAP2A-positive neural crest cells by IF on D8 (fig. S6, J and K).

### Detection of hPGCLCs in the absence of exogenous BMP

Previous studies indicate that hESCs become competent for germ cell fate, as judged by the expression of EOMES and TBXT. These cells show expression of the critical regulators of hPGCLC fate in response to BMP (*6*–*8*). The specific in vitro approaches may differ (*13*, *37*–*40*), but the induction of hPGCLCs requires exogenous BMP2 or BMP4 (*5*, *6*, *8*, *30*, *41*). We observed the expression of EOMES and TBXT in the transiently induced cells (D0), indicating their potential competence for germ cell fate (Fig. 1B). To investigate the specification of PGCLCs in the hGs, we used an hESC line with a *NANOS3-tdTomato* reporter, which is a definitive marker for hPGCs (*7*).

**Fig. 3.**
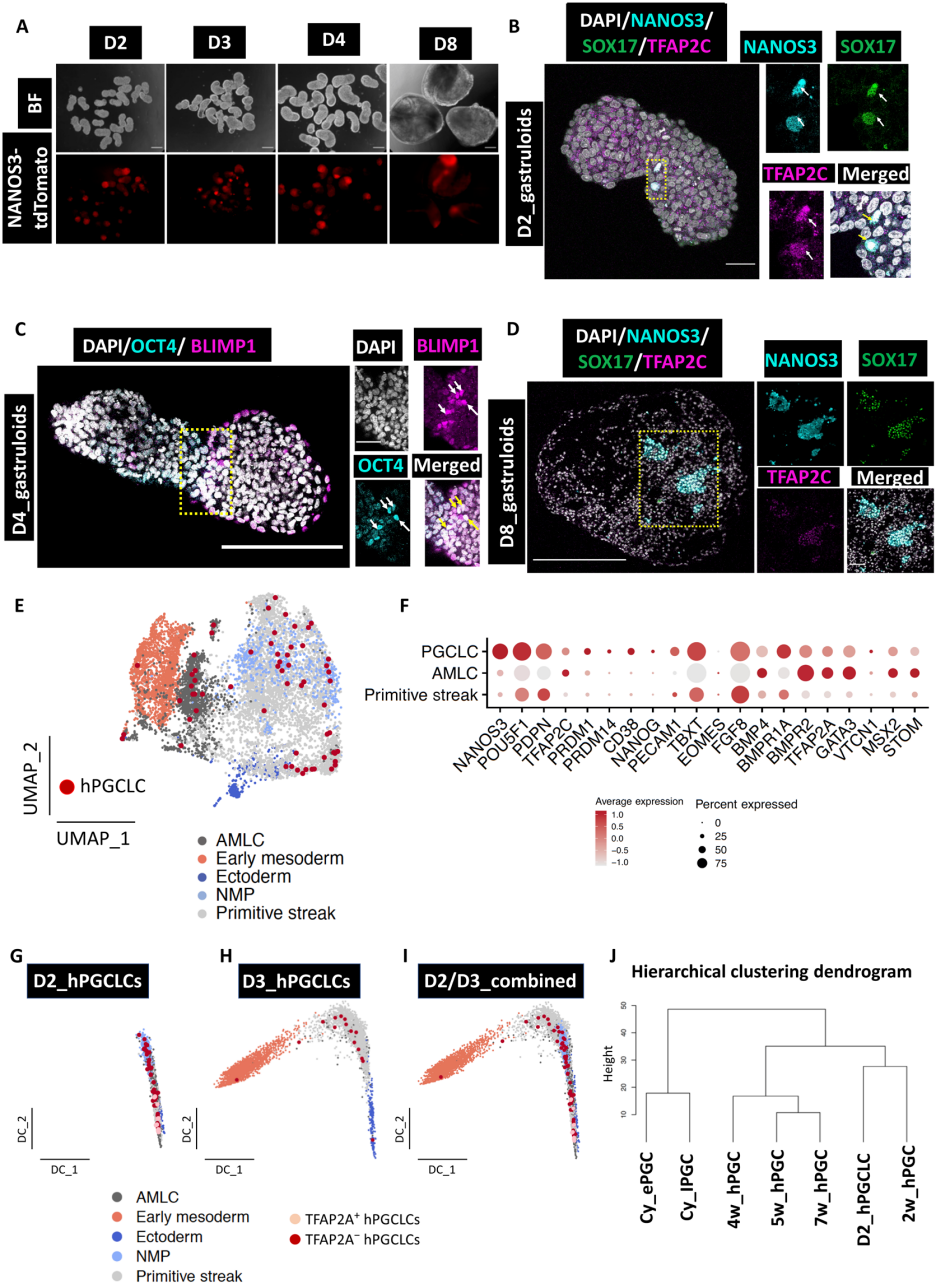
PGCLCs are detected in the absence of exogenous BMP in hGs. (**A**) Microscopic images showing the expression of human PGCLCs in the absence of exogenous BMP, as shown by the NANOS3 reporter in hGs at different time points (*n* = 150 from five experiments). Scale bars, 50 μm. (**B** to **D**) Confocal micrographs showing key hPGCLC markers NANOS3 (RFP), SOX17, and TFAP2C (B); POU5F1 (or OCT4) and BLIMP1 (or PRDM1) (C); and NANOS3 (RFP), SOX17, and TFAP2C (D) by IF in hG sections on D2, D3, and D8, respectively (*n* = 15 from three experiments). Scale bars, 50 μm (B), 100 μm (C), and 250 μm (D). (**E**) UMAP showing the detection of hPGCLCs in the PS and AMLC regions during early specification (D2 and D3 hGs). (**F**) Dot plot showing key hPGCLC markers detected in hGs. BMP4 is specifically enriched in AMLCs. (**G** to **I**) Diffusion components (DC) analysis showing the detection of TFAP2A and TFAP2C on (G) D2 hPGCLCs, (H) D3 hPGCLCs, and (I) D2/D3 combined PGCLC dataset. (**J**) Hierarchical clustering of in vivo PGCs of human and nonhuman primates with in vitro human PGCLCs (D2) from this study. Cy, cynomolgus monkey; hPGCs, human PGCs; w, in vivo developmental stages in weeks; ePGCs, early Cy PGCs; lPGCs, late Cy PGCs.

We first investigated the optimal dosage of the *Wnt* agonist *CH* required for the elongation of hGs along the rostro-caudal axis while ensuring compatibility with hPGCLC specification (*7*). We found that a *CH* concentration of 0.25 μM is optimal for the elongation of hGs and for the specification of hPGCLCs, as judged by the detection of *NANOS3*-*tdTomato* reporter expression from D2, which continued until D8 (Fig. 3A and fig. S7, A and B). Notably, the specification of hPGCLCs occurred without the need for exogenous BMP2/4 supplementation, distinguishing it from other in vitro models (*6*–*8*, *42*). We confirmed our observations for hPGCLC specification by IF on the hG sections on D2, D3, D4, and D8. We observed cells coexpressing established PGC markers: NANOS3^+^SOX17^+^TFAP2C^+^, TFAP2C^+^SOX17^+^POU5F1^+^, and BLIMP1^+^POU5F1^+^ (Fig. 3, B to D, and figs. S7C and S8A). Detection of mouse PGCLCs was recently reported without the addition of exogenous BMP (*43*, *44*).

Next, we analyzed scRNA-seq datasets from D2 and D3 hGs for the putative hPGCLCs. To achieve this, we subclassified PS clusters and reclustered them as PS, AMLC, early mesoderm, ectoderm, and NMP from D2 and D3 hGs (fig. S8, B to D). Because of the rarity of PGCLCs [estimated to be ~60 in pigs; (*7*)] and the potential heterogeneity of gene expression in nascent PGCLCs, we focused on triple-positive cells from D2 and D3 for a combination of key PGC markers: *NANOS3*, *POU5F1*, *PDPN*, *TFAP2C*, *PRDM1*, *PRDM14*, *CD38*, and *NANOG*. Using these criteria, we identified 61 putative hPGCLCs (from *n* = 20 hGs) in the PS and AMLC clusters (Fig. 3E, fig. S8, D and E, and table S1). hPGCLCs on D2 were detected in the AMLCs and PS, whereas on D3, they were only observed in the PS cluster (fig. S8, D and E). Research indicates expression of TFAP2A in the precursors contributing to both the hPGCLCs and AMLC, with a noted loss of *TFAP2A* and a gain of *TFAP2C* occurring in hPGCLCs in the PS (*41*, *45*). In our dataset, we identified putative PGCLCs on D2 showing the coexpression of *TFAP2A* and *TFAP2C.* However, on D3, we only detected *TFAP2C^+^* putative PGCLCs (Fig. 3, G to I). Recent evidence suggests that the PGCs and the amnion share *TFAP2A^+^* precursors (*5*, *45*). Our findings in hGs may clarify and correlate with the detection of certain PGCs in the amnion of monkeys and in human embryo models (*9*, *10*). Trajectory analysis of a rare CS8 human embryo confirmed the shared progenitors for hPGCs and amniotic ectoderm (*18*), similar to our in vitro observation (*45*).

To better characterize hPGCLCs detected in hGs, we compared them with published PGC datasets from human embryos at different developmental stages. In vivo, human PGCs from CS7 human gastrula in weeks 2, 4, 5, and 7 (*46*) as well as early and late PGCs from cynomolgus monkey embryos (*47*) were compared. A hierarchical clustering dendrogram showed that in vitro hPGCLCs from D2 hGs were more similar to the in vivo PGCs from CS7 human gastrula (Fig. 3J). A principal components analysis (PCA) also revealed the transcriptomic similarity of in vitro PGCLCs from D2 hGs to CS7 (week 2) hPGCs compared to later developmental stages (fig. S8F). Despite the transcriptomic closeness of our in vitro hPGCLCs with week 2 hPGCs, they still exhibit transcriptomic differences (fig. S8G). This might be due to the mismatch in the developmental stage between in vitro and in vivo PGCs as we speculate that CS7 hPGCs (approximately D16 to D19), which are the earliest PGCs available, might be more advanced than the nascent PGCLCs. The presence of TFAP2A^+^ or TFAP2A^−^ PGCLCs in the hGs within the same time point (D2) or different time points (D3) suggests a certain level of transcriptional heterogeneity among nascent hPGCLCs. This is rather due to the specification of hPGCLCs in hGs in response to endogenous BMP levels, which are probably much lower than the exogenous BMP dosage normally used to induce hPGCLCs in vitro (*45*). Comparison of PGCLCs in hGs with the published in vitro dataset (*45*) revealed similarity with the earliest hPGCLCs detected between 18- and 24-hour inductions in an embryoid body model (fig. S8H). Notably, at this point, the earliest hPGCLCs diverged from a progenitor population subsequently expressing NANOS3 (*45*), a key identifying PGCLC marker. Hence, these results confirm that PGCLCs detected in hGs are earliest PGCLCs during the time of specification.

### A mutation in *ISL1* in gastruloids affects hPGCLC specification

**Fig. 4.**
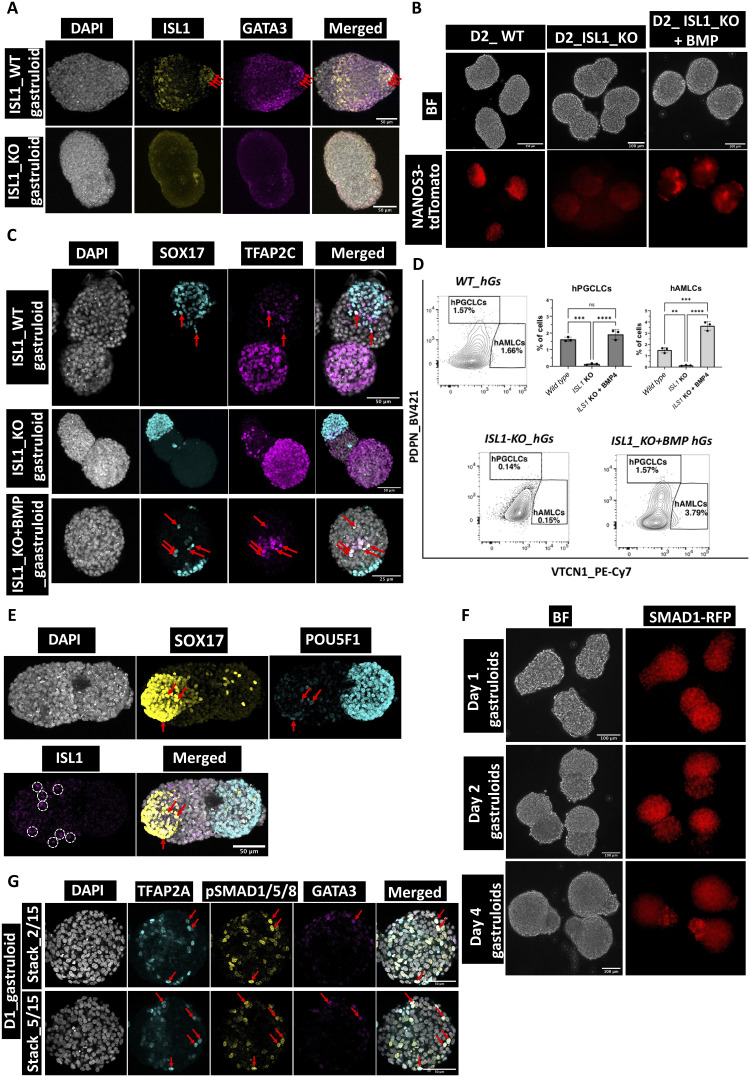
ISL1 KO hGs do not display PGCLC formation. (**A**) Confocal micrographs showing the lack of amnion markers ISL1 and GATA3 in the ISL1 KO hGs. Scale bars, 50 μm (*n* = 10 samples from three experiments). Red arrowheads indicate AMLCs. (**B**) Live microscopic images showing the detection of the fluorescence reporter NANOS3-tdTomato in WT but absent in the ISL1 KO hGs. NANOS3-tdTomato is rescued in gastruloids treated with exogenous BMP supplementation. Scale bars, 250 μm (WT) and 100 μm (KO and KO + BMP) (*n* = 300 samples from five experiments). (**C**) Confocal micrographs showing the maximum intensity projection of PGCLC markers, including SOX17 and TFAP2C in WT, KO, and KO + BMP–supplemented hGs. Scale bars, 50 μm (WT and KO) and 25 μm (KO + BMP) (*n* = 30 samples from three experiments). Red arrowheads indicate hPGCLCs. (**D**) Flow cytometry analysis showing the quantification of PGCLCs and AMLCs in the WT, ISL1 KO, and KO + BMP–supplemented hGs (*n* = 3 samples from three experiments). One-way ANOVA with Tukey’s multiple comparisons test was performed for statistical analysis. *P* < 0.05 was considered significant. ****P < 0.0001, ***P < 0.0005, and **P < 0.005. (**E**) Confocal micrographs with maximum intensity projection of SOX17, POU5F1 (OCT4), and ISL1 showing the distribution of PGCLCs and AMLCs in hGs. PGCLCs are indicated by red arrowheads. AMLCs are shown in white circles. Scale bars, 50 μm (*n* = 15 samples from three experiments). (**F**) Live microscopic images showing the detection of the fluorescence reporter SMAD1-RFP in hGs from D1 to D4. Scale bars, 100 μm (*n* = 300 samples from five experiments). (**G**) Confocal micrographs showing the expression of amniotic ectoderm markers, TFAP2A and GATA3, and pSMAD1/5/8, an intracellular target of BMP4 signaling in D1 hGs. Scale bars, 50 μm (WT). Red arrowheads indicate coexpression of pSMAD1 with either TFAP2A or GATA3 (*n* = 30 samples from three experiments).

A recent study investigating the human PSC–based 3D embryonic model has highlighted the role of ISL1 during hPGCLC specification in vitro (*16*). Previous research on nonhuman primate embryos identified ISL1 as a marker of AMLCs (*48*, *49*). In our observations of hGs, we detected ISL1^+^ cells as early as D1 of aggregation (Fig. 4A) (*16*). In addition, NANOS3^+^ hPGCLCs were detected as early as D2 in these hGs (Fig. 4B and fig. S12A).

To further investigate the role of the amniotic marker ISL1 in hPGCLC specification, we generated ISL1 knockout (KO) hESCs using CRISPR-Cas9–based gene editing (fig. S9, A and B). The loss of ISl1 resulted in the absence of another AMLC marker, GATA3, in hGs (Fig. 4A). We allowed ISL1 KO hESCs to self-organize into 3D gastruloids but did not detect PGCLCs, as NANOS3 was absent in ISL1 mutant gastruloids (Fig. 4B and fig. S9, C to E).

We hypothesized that the BMP signal is absent in ISL1 KO gastruloids, which prevents the formation of hPGCLCs. To test this hypothesis, we supplied exogenous BMP2 (100 ng/ml) in the culture medium and allowed the formation of gastruloids, which remarkably rescued the NANOS3^+^ hPGCLCs (Fig. 4B and fig. S9E). Together, these observations show that AMLC is a source of endogenous BMP signal, which induces hPGCLCs, consistent with a recent study (*16*).

To verify the hPGCLC identity, we performed IF for key PGCLC markers, including SOX17 and TFAP2C. We observed that while SOX17 and TFAP2C were expressed separately in the ISL1 KO hGs, hPGCLCs were not detected (Fig. 4C). However, when BMP2 was supplemented to the culture medium, the presence of hPGCLCs was restored, similar to the wild-type (WT) condition (Fig. 4C). These results further demonstrate that AMLC is the source of endogenous BMP, which induces hPGCLCs.

Next, using flow cytometry, we quantified the number of hPGCLCs and AMLCs in D2 hGs. Our analysis revealed significantly lower percentages of AMLC (<0.5%) and PGCLC (<0.5%) in the ISL1 KO hGs compared to the WT and ISL1 KO + BMP gastruloids (Fig. 4D and fig. S10). These findings further confirm the role of AMLCs as the source of BMP in the specification of hPGCLCs. Overall, the number of PGCLCs in hGs is low, consistent with our observation by scRNA-seq and IF (Fig. 3, B to E). AMLCs are detected earlier than PGCLCs and may serve as a source of endogenous BMP signaling to induce PGCLCs in hGs. We also investigated the distribution of PGCLCs and AMLCs in the hGs. While ISL1^+^ cells were in proximity to SOX17^+^ endodermal cells, they were not colocalized with SOX17^+^POU5F1^+^ double-positive hPGCLCs (Fig. 4E and fig. S11A), similar to a recent report (*16*).

We next investigated the presence of SMAD1, an intracellular signaling molecule activated by BMP4. We used RUES2 hESCs with an SMAD1-RFP reporter (*50*) and detected SMAD1 already in D1 during the formation of gastruloids (Fig. 4F). We detected phosphorylated SMAD1/5/8 (pSMAD1/5/8) together with amnion markers TFAP2A and GATA3 in D1 gastruloids (Fig. 4G). Notably, TFAP2A was however present in ISL1 KO gastruloids, although GATA3 was absent (Fig. 4G and fig. S11B). GATA3 was restored upon exogenous BMP supplementation to ISl1 KO gastruloids (fig. S11B). These findings confirm that BMP signaling is present from D1, likely through WNT signaling–mediated BRA activation (fig. S1C) (*51*). This pathway may induce the formation of AMLCs in hGs.

We then examined the chemical inhibition of WNT signaling by using IWP2 on intracellular response to BMP activation. IWP2 inhibits SMAD1 detection as well as the specification of PGCLCs in gastruloids (fig. S12, B and C). This suggests that early activation of *WNT* induces AMLCs, which subsequently promotes specification of PGCLCs in gastruloids. Our scRNA-seq data further indicate the role of BMP4, which is contributed by mixed mesoderm detected in D2 gastruloids (fig. S12D). This is in line with a previous study in 2D gastruloids showing a signaling cascade: Accordingly, *WNT* signaling activates *NODAL* signaling, which in turn activates BMP signaling (*52*).

Last, we examined the influence of *WNT* and *ACTIVIN* signaling pathways on inducing competence for the germline fate in hGs. To do this, we allowed self-organization of hESCs treated with *CHIRON* alone or combined with *ACTIVIN*. Notably, the absence of Activin during mesendoderm preinduction resulted in a failure to form PGCLCs (fig. S12E). Activin activates the *NODAL* signaling pathway, which, together with *WNT* signaling, ensures competence for the germline fate through activation of EOMES and Brachyury—key factors for PGCLC specification (fig. S12, F and G) (*6*, *7*). This supports recent research highlighting the role of *NODAL* signaling in inducing the PGC competent state (*53*). In summary, our findings indicate that *WNT* and *NODAL* signaling establishes the foundation for the germline competent state in hGs. Furthermore, early mesodermal cells activate *BMP* signaling that induces AMLCs, which trigger PGCLC induction in hGs.

## DISCUSSION

Our study confirms that hESCs can self-organize into 3D gastruloids (hGs) that elongate along a rostro-caudal axis and form all three germ layers (*27*, *43*, *44*). These hGs display postgastrulation features, which include cardiomyocytes and NMPs. The gene expression patterns observed in hGs on D3 align with the development stage of CS7 human embryos, offering insights into early human development.

Notably, we observed hPGCLCs without exogenous BMP and provide evidence suggesting that AMLCs may be a source of endogenous BMP necessary for PGCLC specification. Detecting putative hPGCLCs within AMLC and PS-like cells in hGs may suggest a dual origin of nascent PGCLCs (*54*). However, the PGCLCs associated with PS-like cells later showed a loss of TFAP2A, which is indicative of definitive hPGCLCs (*5*, *45*). Direct observation of authentic ex vivo early human embryos in culture beyond 14 days is essential to validate our conclusions whenever possible.

The development of both AMLC and PGCLC is closely linked to BMP signaling (*14*, *16*, *48*). Our findings indicate that the loss of ISL1 results in the absence of both AMLCs and PGCLCs. The addition of exogenous BMP to the ISL1 mutant hGs restored the formation of PGCLCs. This indicates that AMLC is a likely source of BMP in WT hGs. We propose that WNT-activated mesodermal cells provide BMP signaling that induces AMLCs, which, in turn, serve as a source of BMP for the specification of PGCLCs in hGs.

Our model is a reproducible, self-organized, nonintegrated stem cell–based gastruloid (hGs) that has the potential to investigate key aspects of human gastrulation and postgastrulation development. With further improvements, this model could be advanced to explore later stages of human development in culture, including genetic screenings related to human development and diseases.

The formation of hGs involves inducing PSCs to exit from pluripotency, generating heterogeneity, and enabling their self-organization. We detected the emergence of some cell types, including hPGCLCs in the hGs, but analyzing rare cells by scRNA-seq was challenging. The PGCLCs in the hGs remained nascent, with little proliferation or migration over 8 days. As the size of the hGs increased, the dilution of PGCLCs made them harder to detect by scRNA-seq. Focus on nascent or early PGC specification, optimizing experimental design and culture conditions, could enhance the structural features and spatial similarity to in vivo human embryo development.

## MATERIALS AND METHODS

### Ethics statement

The use of hESCs for this study was approved by the Human Biology Research Ethics Committee of the University of Cambridge, UK. All experiments were conducted under the relevant guidelines and regulations for using hESCs and in compliance with the ISSCR 2021 guidelines to generate hGs. The use of cell lines to generate hGs was peer reviewed and approved as part of the Wellcome Human Development Biology Initiative (HDBI) grant application (G112785). No ethical approval is required in the UK for studies using hESCs to generate hGs. Together, this investigation falls within the existing legislation set by regulatory bodies in the UK. The Gurdon Institute Safety committee carried out appropriate scrutiny, including risk assessments. This study did not involve the use of human preimplantation embryos.

### hESC culture and induction of transient state

We used *W15-NANOS3-tdTomato*, *WIS2* and *RUES2* hESC lines in this study. All hESCs were cultured in Essential 8 Medium (Life Technologies, A1517001) on freshly prepared vitronectin (Thermo Fisher Scientific, A31804)–coated six-well plates in humidified chambers at 37°C and 5% CO_2_. hESCs were passaged every 3 to 4 days (once they reached ~70% confluency) using 0.5 mM EDTA (Thermo Fisher Scientific, AM9260G) in homemade 1× phosphate-buffered saline (PBS). The transient state of cells was induced as described previously, with slight modifications (*7*). Briefly, hESC colonies (70% confluent) were dissociated into single cells using 0.25% trypsin-EDTA (Life Technologies, 25200072), seeded (600,000 cells per well) into freshly prepared vitronectin-coated six-well plates (~30 min), and cultured for 24 hours in the presence of Advanced RPMI 1640 (Life Technologies, 61870-036) supplemented with B27 supplement (1%) (Thermo Fisher Scientific, 17504044), 0.1 mM nonessential amino acids, penicillin (100 U/ml), streptomycin (0.1 mg/ml), 2 mM l-glutamine, Activin A (1 μg/ml) (in-house produced by Biochemistry Department, University of Cambridge), Chiron (3 μM) (CHIR99021, TOCRIS 4423/10), and Rho-associated, coiled coil–containing protein kinase inhibitor (10 μM; Y27632) (hereafter called induction medium).

### Self-organization of hESCs into 3D hGs

Transient cells induced in induction medium for 24 hours were dissociated into single cells using 0.25% trypsin-EDTA and seeded (400 cells per well) into Corning Clear ultralow attachment 96-well plates (Thermo Fisher Scientific, 10023683). Essential 6 medium (100 μl per well; Life Technologies, A1516401) supplemented with 0.25 μM *CH* and Rho-associated, coiled coil–containing protein kinase inhibitor (10 μM) was used for self-organization of the intermediate cells [hereafter termed as organization medium (OM)]. Ninety-six–well plates with singly dissociated cells were centrifuged at 1200 rpm for 5 min to let the cells settle at the center bottom of each well. Peripheral wells of the 96-well plates were filled with the same volume of 1× PBS to avoid evaporation of the culture medium. PBS was added in peripheral wells of the plate also to avoid scattering of cells in outer wells even after centrifugation that would otherwise form satellite aggregates instead of forming a single aggregate during self-organization. *CH* (0.25 μM) for 100 μl of OM per well was used for the first 3 days of self-organization. On D3, 100 μl of Essential 6 medium was added to each well, resulting in ~0.125 μM *CH* in a volume of 200 μl of OM, and the hGs were cultured for the next 3 days. On D6, 100 μl of OM was removed from each well and 100 μl of Essential 6 medium was added to them, resulting in ~0.06 μM *CH* in the final 200 μl of OM, in which hGs were cultured for additional 2 days (total of 8 days).

### IF of hESCs and transiently induced cells

hESCs and transiently induced cells were cultured on Ibidi eight-well μ-slides (Thistle Scientific Ltd., IB-80826). Transiently induced cells cultured for 24 hours were washed in 1× PBS and fixed in 4% paraformaldehyde (PFA) for 10 min at room temperature, whereas hESCs were cultured for 3 to 4 days before fixation. Samples were incubated with primary antibodies overnight at 4°C and with fluorescently conjugated secondary antibodies for 1 hour at room temperature. Samples were stained with 4′,6-diamidino-2-phenylindole (DAPI; Sigma-Aldrich, D9542-1MG) to mark nuclei and were observed under confocal laser scanning microscopy. Antibodies used here are listed in table S1.

### Cryosectioning and IF

hGs cultured on ultralow attachment 96-well plates were collected, washed in 1× PBS, and fixed in 4% PFA for 3 to 4 hours at room temperature or 1% PFA overnight at 4°C. hGs were embedded in optimal cutting temperature compound (OCT) mounting medium (VWR-361603E) and incubated in dry ice for 30 min before sectioning the frozen samples. The antigen retrieval procedure was performed to remove the fluorescence reporter from the *W15-NANOS3-tdTomato* hESC line, where required. Briefly, cryosectioned slides were incubated at room temperature for 30 min and boiled in tris-EDTA buffer (pH 6.0) for 30 min in a microwave oven. Cryosectioned slides were dried up at room temperature for 45 min before performing IF. The anti-RFP primary antibody was used to visualize *NANOS3* protein in the *W15-NANOS3-tdTomato* hESC line, where required. Antigen retrieval was performed to remove the endogenous tagging, where required. All samples were incubated with primary antibodies overnight at 4°C and with fluorescently conjugated secondary antibodies for 1 hour at room temperature. Samples were stained with DAPI (Sigma-Aldrich, D9542-1MG) to mark nuclei and were observed under confocal laser scanning microscopy. Antibodies used here are listed in table S1.

### Image analysis

IF was followed by confocal microscopy (Leica SP8 and SP5 inverted microscope) for imaging. Image analysis was carried out by the image processing package Fiji (*55*). For 2D image analysis and quantification (fig. S12G), we developed a custom script for Fiji (*55*) to segment nuclei using a frequency bandpass filter and measure their (DAPI, POU5F1, EOMES, and BRA) labeling intensity. For 3D image analysis and quantification (Fig. 1F), we used a custom script for Fiji (*55*) to generate volume masks in each channel to measure the volume of labeling. We calculated the proportion of the gastruloid volume defined by DAPI labeling that was positive for each marker (namely, SOX17, SOX2, and BRA) and double positive for SOX2 and BRA.

### Generation of ISL1 KO hESCs

We generated ISl1 KO hESCs in the W15-Nanos3-tdTomato reporter cell line (*7*) using CRISPR-Cas9–based gene editing. Briefly, guide RNAs (gRNAs) targeting exon 1 and exon 2 of the ISL1 gene were selected on the basis of a previously published study (*48*). gRNA targeting exon 1 (forward strand): caccgCTTACAGATATGGGAGACAT; gRNA targeting exon 1 (reverse strand): aaacATGTCTCCCATATCTGTAAGc; gRNA targeting exon 2 (forward strand): caccgTGCGGCATGTTTGAAATGTG; gRNA targeting exon 2 (reverse strand): aaacCACATTTCAAACATGCCGCAc.

The pX330-U6-Chimeric_BB-CBh-hSpCas9 plasmid was used a vector, where gRNAs were cloned and inserted into hESCs using nucleofection. Forty-four hESC colonies were manually picked, amplified, and genotyped to identify KO clones. Two KO clones (nos. 13 and 25) were selected and confirmed by agarose gel electrophoresis (fig. S9B) followed by Sanger sequencing (fig. S9A). Two WT clones were selected as controls. All KO studies were performed using these clones.

### Flow cytometry analysis

Quantitative flow cytometry was performed on a SONY SH800 cell sorter. Fluorescence-activated cell sorting (FACS) gates of live single cells of the correct scatter and size were stringently set after compensation with individual fluorescence controls for each channel to distinguish true positive populations from autofluorescence or fluorescence spillover. Percentages shown are percentage of parent population, with the gating strategy listed in all corresponding figures (fig. S10). Briefly, hGs were dissociated in 0.25% trypsin-EDTA (GIBCO) for ~15 min at 37°C, quenched in 10% fetal bovine serum, centrifuged for 2 min at 700 rpm, resuspended in 2% fetal bovine serum, and filtered through a 70-μm mesh. Cells were then stained with PDPN (BV421), VTCN1 (PE-Cy7), and DAPI for 30 min at room temperature in a rotating mixture. WT, ISL1 KO, and three ISL1 KO supplemented with BMP4 (100 nM) were analyzed from three experiments. Twenty hGs were used for each condition. Data were analyzed, and plots were generated using FlowJo version 10.8.2.

### 10× genomics

For each stage, 20 hGs were collected and dissociated into single cells using 0.5% trypsin-EDTA. Singly dissociated cells were collected into an Eppendorf tube containing PBS with 0.04% (w/v) bovine serum albumin (400 μg/ml). Cells were filtered using 50 μM disposable filters (CellTrics) and counted with an automatic cell counter (LUNA dual fluorescence cell counter). Cells were loaded into the 10× Genomics Chromium using the Single Cell 3′ Reagents Kit version 3. Libraries were prepared as per the manufacturer’s instructions and pooled for sequencing. Libraries were sequenced on NovaSeq 6000. Sequencing was performed in biological replicates for each stage derived from independent experiments.

### scRNA-seq analysis

The reference genome was generated with command mkref from CellRanger (version 4.0.0) using prebuilt human reference transcriptomes (GRCh38). Starting from fastq files, raw count matrices were obtained with the CellRanger count for each of the two batches per sample. Quality control analysis was performed with Seurat (version 4.0.5) (*56*) on the raw count matrices, keeping only cells with the number of expressed genes greater or equal to 1000 and the fraction of mitochondrial genes below or equal to 0.20. This resulted in a total of 11,983 cells on D0, 8590 on D2, 7751 on D3, 18,494 on D4, and 23,593 on D8. An additional filtering step was performed with SoupX to take potential environmental RNA contamination into account. After normalization with function NormalizeData in Seurat (*56*) and correcting for batch effects with the FindIntegrationAnchors function (reduction = “cca”), cluster analysis was performed with the FindClusters function (resolution = 0.1) on the top 20 PCA components computed from the top 2000 genes selected with SelectIntegrationFeatures. The optimal resolution value was chosen according to the output of the clustree function (version 0.4.4) (*57*). To improve sensitivity to small clusters, cluster analysis at each time point was refined using the concept of entropy of mixing. Briefly, we selected genes frequently detected in small cell neighborhoods by ranking them according to their entropy of mixing computed in local regions defined from the kNN graph. Then, sub-clustering was performed on clusters enriched with low-entropy genes, verified by Fisher’s test. This analysis resulted in one additional cluster on D2 and two additional clusters on D8.

Marker analysis for each cluster was performed with FindMarkers in Seurat using default parameters. Only markers with adjusted *P* value (Bonferroni correction) below or equal to 0.05 were considered for downstream analysis. Last, for each cluster, only unique markers that were not included in the marker list of other clusters were used for further analysis. Clusters were annotated by comparing key markers with CS7 human gastrula (*4*) and mouse embryos (*58*) as well as selecting them manually from the list of markers identified by FindMarkers for each cluster at each time point. For visualizing all the clusters from all days together (apart from D0), first, batch correction between samples from different time points was done with Scanorama (*59*) (function correct_scanpy with return_dimred = True). Batch integration was done using, as features, all the genes that were variable in at least two different days with scanpy (*60*) (version 1.8.0) [function pp.highly_variable_genes (with parameter batch_key = “day”) and then scanpy functions pp.neighbors (with parameters n_pcs = 50 and use_rep = “X_scanorama”), tl.paga (with default parameters), and pl.paga (with parameters edge_width_scale = 0.05 and threshold = 0.4)]. UMAP plot visualization was then generated with scanpy functions tl.umap (with parameter init_pos = “paga”) and pl.umap.m.

On D2 and D3, a subcluster analysis was performed starting from the PS cluster (with FindClusters function with resolution = 0.1). The analysis resulted in three additional clusters that were labeled as PS 1, PS 2, and NMPs.

The projection of scRNA-seq data onto CS7 human gastrula was done with the function “FindTransferAnchors” (Seurat version 4.0.5, with parameters k.anchor = 100, k.filter = 500, and reduction = “pcaproject”) using, as a reference, the CS7 human embryo and, as a query, the hGs at different time points. Cell labeling was performed with the “TransferData” function (Seurat version 4.0.5, with parameter k.weight = 100). For each cluster from hGs at a given time point, the prediction score was defined as the average of maximum score assigned to each cell (given by “TransferData”).

To compare our gastruloids with publicly available scRNA-seq datasets, raw counts were obtained from Gene Expression Omnibus [accession: GSE123187, hGs (*27*); GSE193007, primate gastrulation (*32*)] and reanalyzed as described above. Cluster and cell type annotations were obtained from tables S1 and S2. Processed and annotated public scRNA-seq datasets were down-sampled to 10,000 cells to make them comparable and integrated with our combined gastruloid scRNA-seq datasets using reciprocal PCA, as implemented in the FindIntegrationAnchors function in Seurat (reduction = “rpca”) and visualized by UMAP. To quantify cell type similarity independently of UMAP, the FindTransferAnchors function using the top 15 principal components and the TransferData function in Seurat were used to predict cell types in our gastruloids using the public dataset as a reference; average prediction scores were shown as heatmaps.

To test whether we could find a rostral-caudal signal in the mesodermal cells from our gastruloids, we took the clusters from D3 that resembled the advanced mesoderm cluster in the CS7 dataset (on the basis of the cell type scores introduced above), where the transcriptional differences between rostral and caudal genes were the strongest (*61*). In particular, we took the human embryonic organoid clusters annotated as intermediate mesoderm and mixed mesoderm, because most of the cells from them were mapped onto the CS7 advanced mesoderm cluster. We trained a Seurat classifier on the caudal versus rostral cells from the CS7 advanced mesoderm cluster and then used it to classify the human embryonic organoid cells as caudal or rostral. A Wilcoxon rank-sum test was used to find differentially expressed genes between the caudal and rostral cells.

Diffusion maps were generated from specific subsets of clusters in the dataset (figs. S5 and S6) using the Scanorama-integrated gene expression data as follows: 1000 highly variable genes were selected using the function FindVariableFeatures from the Seurat library in R. Then, on the basis of these genes, a *k*-nearest neighbor graph was calculated using the function pp.neighbors on 15 principal components specifying that Scanorama-integrated data should be used (n_pcs =15, use_rep = “X_scanorama”). Last, the diffusion map was generated on the same 15 components with the function tl.diffmap with parameter n_comps = 15. Raw data for the hGs are available through ArrayExpress under accession number E-MTAB-12045.

### Identification of hPGCLCs from scRNA-seq

The primitive streak, NMP, ectoderm, and early mesoderm clusters from D2 and D3 were integrated using canonical correlation analysis, as implemented in the FindIntegrationAnchors function in Seurat (reduction = “cca”). Cells were reclustered using the Louvain algorithm on the *k*-nearest neighbor graph calculated for the top 15 principal components with a resolution of 1.0. One subcluster was identified as AMLC expressing the markers *TFAP2A*, *GATA3*, *MSX2*, and *STOM*. Sixty-one putative PGCLCs were identified on D2 and D3 as triple-positive cells (normalized counts >0.1) of key PGC markers among *NANOS3*, *PDPN*, *POU5F1* (or *OCT4*), *TFAP2C*, *PRDM1*, *PRDM14*, and *CD38*. Only potential PGCLC combinations were selected for further analysis (see table S1). hPGCLCs with coexpression of *TFAP2A* and *TFAP2C* (normalized counts >0.1) are highlighted. To compare our PGCLCs with publicly available scRNA-seq datasets, raw counts for 18- to 48-hour and D4 PGCLCs were obtained from Castillo-Venzor *et al.* (*45*), normalized using Seurat, and merged with the normalized counts of our 61 putative PGCLCs. UMAP and PCA were performed in Seurat.

### Comparison between hPGCLCs and published datasets from in vivo PGCs

hPGCLCs from D2 were compared with published PGC datasets from human embryos at time points of 2 weeks (*4*), 4 weeks, 5 weeks, and 7 weeks (*46*). A comparison was also made with early and late PGCs from cynomolgus monkey embryos (*47*). Early PGCs were mainly derived from the embryo stages E16 and E17, while late PGCs were primarily from stages E43, E50, and E51.

Because the published human and monkey datasets provide different normalized count matrices (transcripts per million for human PGCs and reads per million for monkey PGCs), quantile normalization was performed on the normalized counts of hPGCLCs, human PGCs, and monkey PGCs using the normalizeQuantiles function from the limma R package (*62*). Markers for each time point in the human and monkey datasets were identified using the FindMarkers function in Seurat (*56*) with default parameters (refer to the “scRNA-seq analysis” section).

Hierarchical clustering was performed by constructing a dendrogram using the hclust function in R (*63*), with the union of the identified markers as features. The clustering was based on the Euclidean distance between the average expression profiles of the in vivo time points and hPGCLCs. A heatmap of the PGC markers used to identify hPGCLCs was generated using the heatmap.2 function from the gplots R package (*64*).

PCA was conducted starting with human PGCs using the markers identified at each time point as features. The prcomp function was used with arguments center = TRUE and scale. = TRUE. The hPGCLCs were projected onto this existing PCA space using the predict function in R.

The expression patterns of *BMP4* and its receptor *BMPR1B* were investigated on D2 using the cluster partition that includes AMLC and PGC (refer to the “Identification of hPGCLCs from scRNA-seq” section). The mean expression of *BMP4* across all clusters and *BMPR1B* in the PGC cluster was computed, and their product was used as a weight to represent the connection strength between clusters. The top four links were retained and visualized as a network using the plot.igraph function from the igraph R package (*65*). The following markers were used to identify each cluster. AMLCs: “*GATA3*,” “*TFAP2A*,” “*TFAP2C*,” “*MSX2*,” and “*STOM*”; mixed mesoderm: “*TBXT*,” “*PDGFRA*,” “*TMEM88*,” “*MESP1*,” “*MESP2*,” “*KDR*,” and “*MSGN1*”; PGCLCs: “*PDPN*,” “*NANOS3*,” “*PRDM1*,” “*CD38*,” and “*PECAM1*.”

### Statistical analysis

To compare variability within and between groups, we used the standard one-way analysis of variance (ANOVA) test for statistical analysis using GraphPad Prism (version 10.1.2).
