# Supplementary material for: The emergence of human primordial germ cell–like cells in stem cell–derived gastruloids
Source: Sci Adv. 2025 Mar 26;11(13):eado1350. doi: 10.1126/sciadv.ado1350 (PMC11939039; doi:10.1126/sciadv.ado1350)
Supplement: Supplementary file 1 — Figs. S1 to S12 Tables S1 and S2 Legend for movie S1 [file sciadv.ado1350_sm.pdf]

Science Advances
AAAS

Supplementary Materials for

The emergence of human primordial germ cell–like cells in stem cell–derived gastruloids

Jitesh Neupane *et al.*

Corresponding author: Jitesh Neupane, jn418@cam.ac.uk; M. Azim Surani, a.surani@gurdon.cam.ac.uk; Antonio Scialdone, antonio.scialdone@helmholtz-munich.de; Sabine Dietmann, sdietmann@wustl.edu

Sci. Adv. **10**, eado1350 (2025)
DOI: 10.1126/sciadv.ado1350

The PDF file includes:

Figs. S1 to S12
Tables S1 and S2
Legend for movie S1

Other Supplementary Material for this manuscript includes the following:

Movie S1

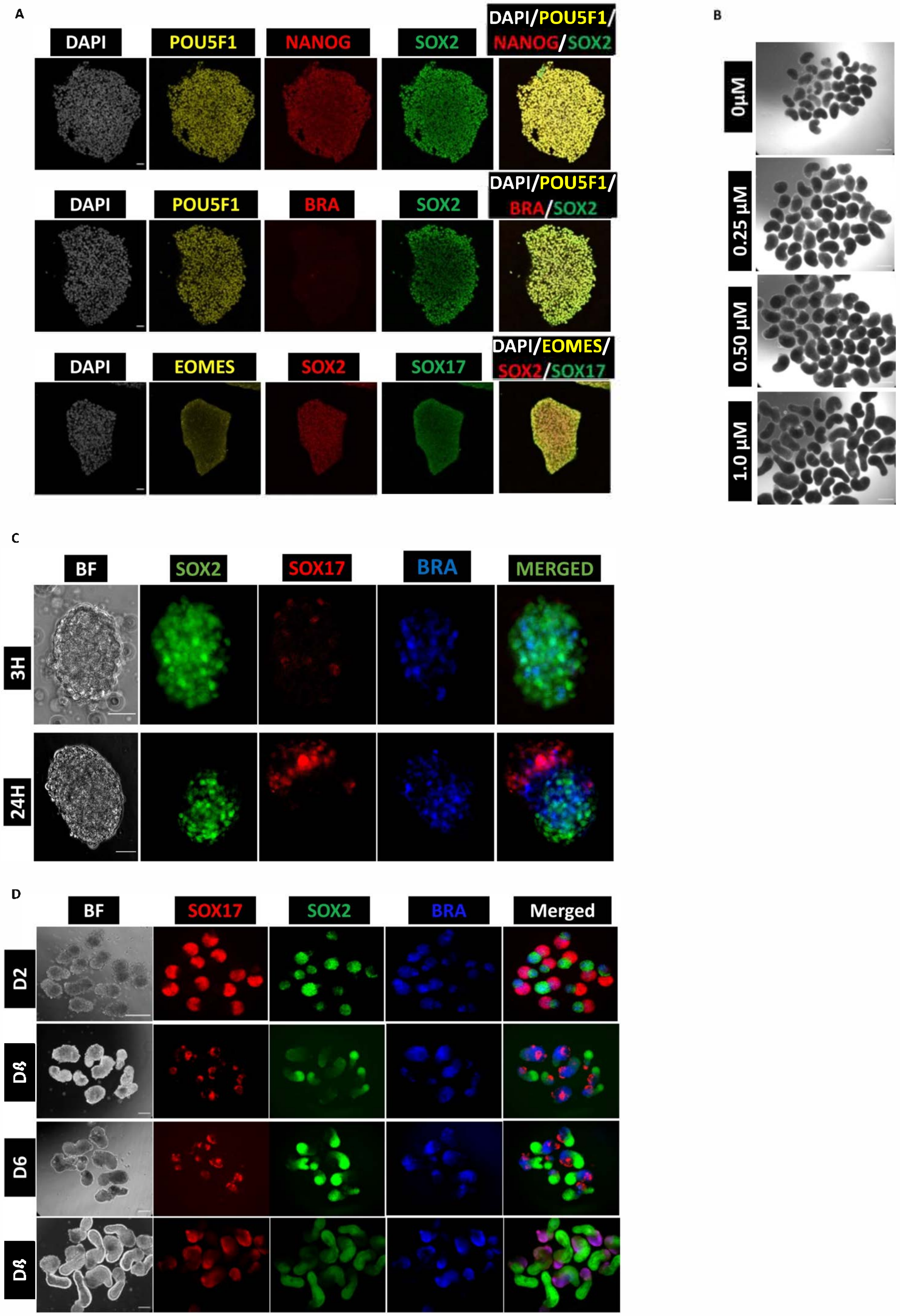
A
DAPI
POU5F1
NANOG
SOX2
DAPI/POU5F1/NANOG/SOX2
BRA
DAPI/POU5F1/BRA/SOX2
EOMES
SOX17
DAPI/EOMES/SOX2/SOX17
B
0μM
0.25 μM
0.50 μM
1.0 μM
C
BF
MERGED
3H
24H
D
Merged
D2
D6

Supplementary Figure 1: Self-organisation of hESCs into human gastruloids (hGs) establishes the three germ layer derivatives.

A, Immunofluorescence staining of W15 hES cells showing the expression of pluripotency markers. Top, *POU5F1* (or *OCT4*), *NANOG* and *SOX2*; Middle, *POU5F1*, *BRA* (or *Brachyury*) and *SOX2*; Bottom, *SOX2, EOMES* and *SOX17. BRA, SOX17 and EOMES* are used as negative marker controls in hESCs. Scale bar, 50µm. **B**, Microscopic images showing the effect of *Wnt* agonist, *Chiron*, on hGs formation. Scale bar, 250µm. **C**, Microscopic images showing the formation of hG aggregates at 3h and 24h. Scale bar, 250µm. **D**, Establishment of the three germ layer derivatives on hGs derived from *RUES2-GLR* hESCs. Reporter colors represent ectoderm (*SOX2-mCitrine*), mesoderm (*BRA-mCerulean*), and endoderm (*SOX17-tdTomato*). Scale bar, 100µm.

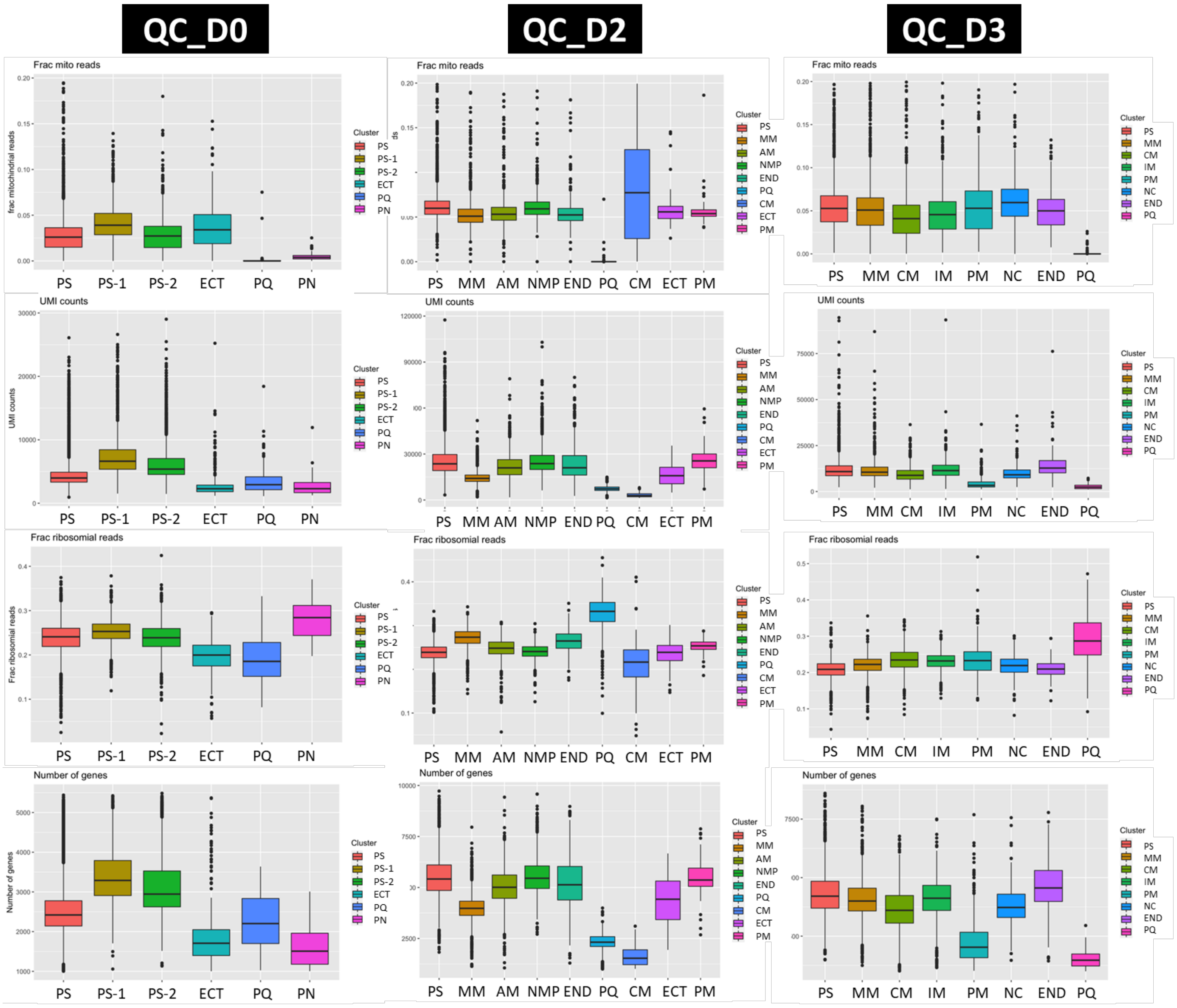
QC_D0
QC_D2
QC_D3
Frac mito reads
frac mitochondrial reads
UMI counts
Frac ribosomial reads
Number of genes
Cluster
PS
PS-1
PS-2
ECT
PQ
PN
MM
AM
NMP
END
CM
PM
IM
NC

Supplementary Figure S2: Quality control of scRNAseq data.

Metrics used to assess the quality of the scRNA libraries from day (D) 0, D2 and D3 samples and clusters. 10X Genomics was performed in replicates (n=2) derived from independent experiments. For each replicate, 20 human gastruloids (hGs) were pooled and loaded into the 10x-Genomics Chromium using the single cell 3' reagents kit v3. From left to right the plots show the fraction of reads mapped to mitochondrial genome, unique molecular identifier counts (or transcripts) per cell, fraction of reads mapped to ribosomal RNA and number of detected genes. Poor quality (PQ) clusters were removed from further analysis. PS-primitive streak, ECT-ectoderm, PN-primitive node, MM-mixed mesoderm, AM-advanced mesoderm, NMP-neuromesodermal progenitors, END-endoderm, CM-cardiac mesoderm, PM-paraxial mesoderm, IM-intermediate mesoderm, NC-neural crest, PQ-poor quality clusters.

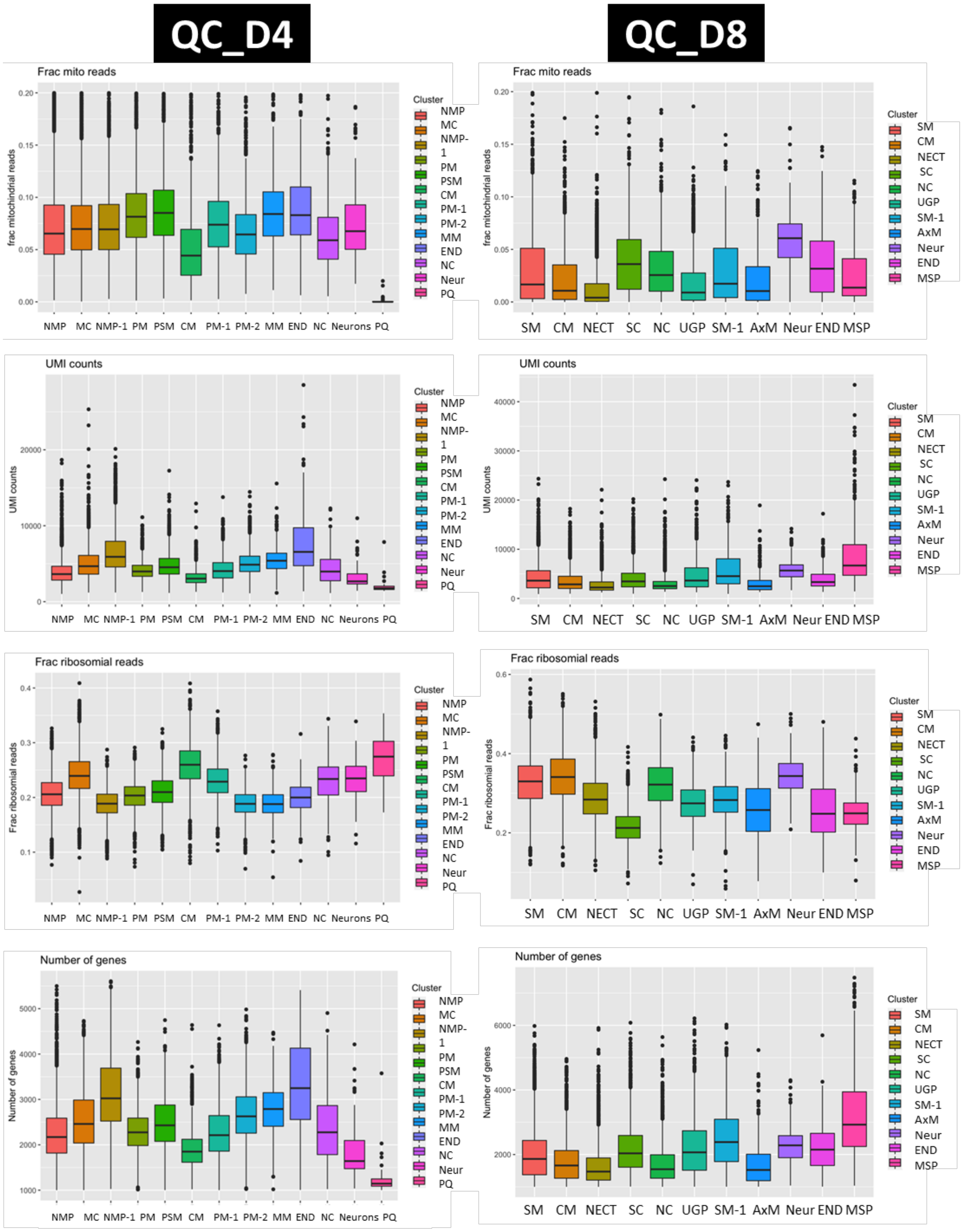

QC_D4
QC_D8
Frac mito reads
frac mitochondrial reads
UMI counts
Frac ribosomial reads
Number of genes
Number of genes
Cluster
NMP MC NMP-1 PM PSM CM PM-1 PM-2 MM END NC Neurons PQ
NMP MC NMP-1 PM PSM CM PM-1 PM-2 MM END NC Neur PQ
SM CM NECT SC NC UGP SM-1 AxM Neur END MSP

Supplementary Figure S3: Quality control of scRNAseq data.

Metrics used to assess the quality of the scRNA libraries from day (D) 4 and D8 samples and clusters. 10X Genomics was performed in replicates derived from independent experiments. For each replicate, 20 human gastruloids (hGs) were pooled and loaded into the 10x-Genomics Chromium using the single cell 3’ reagents kit v3. From left to right the plots show the fraction of reads mapped to mitochondrial genome, unique molecular identifier counts (or transcripts) per cell, fraction of reads mapped to ribosomal RNA and number of detected genes. Poor quality (PQ) clusters were removed from further analysis. MM-mixed mesoderm, NMP-neuromesodermal progenitors, END-endoderm, CM-cardiac mesoderm, PM-paraxial mesoderm, PSM-presomitic mesoderm, NC-neural crest, NECT-neural ectoderm, SM-somatic mesoderm, UGP-urogenital progenitors, AxM-axial mesoderm, MSP-musculoskeletal precursors, SC-spinal cord, Neur-Neurons.

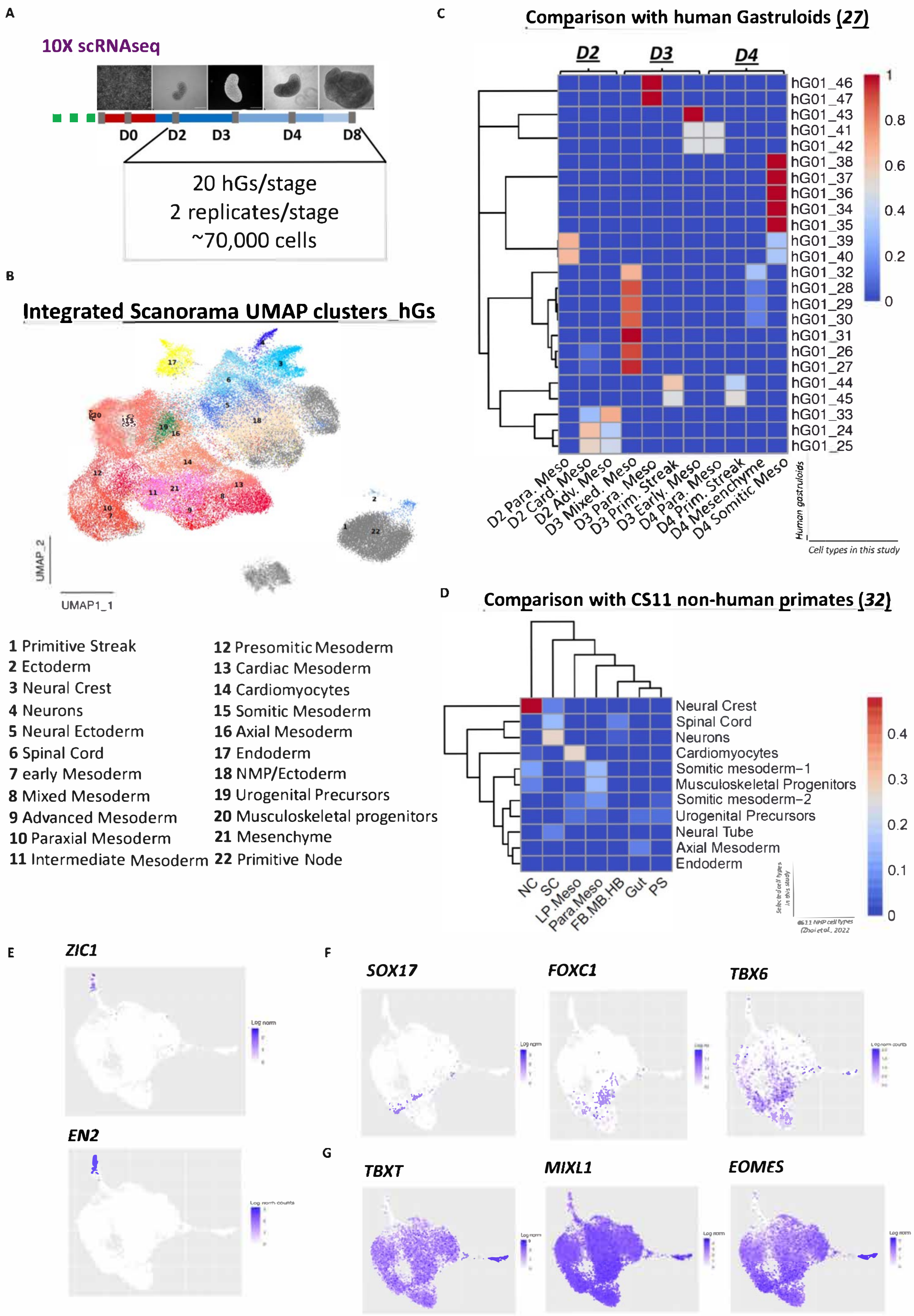
A
10X scRNAseq
D0
D2
D3
D4
D8
20 hGs/stage
2 replicates/stage
~70,000 cells
B
Integrated Scanorama UMAP clusters_hGs
UMAP_2
UMAP1_1
1 Primitive Streak
2 Ectoderm
3 Neural Crest
4 Neurons
5 Neural Ectoderm
6 Spinal Cord
7 early Mesoderm
8 Mixed Mesoderm
9 Advanced Mesoderm
10 Paraxial Mesoderm
11 Intermediate Mesoderm
12 Presomitic Mesoderm
13 Cardiac Mesoderm
14 Cardiomyocytes
15 Somitic Mesoderm
16 Axial Mesoderm
17 Endoderm
18 NMP/Ectoderm
19 Urogenital Precursors
20 Musculoskeletal progenitors
21 Mesenchyme
22 Primitive Node
C
Comparison with human Gastruloids (27)
D2
D3
D4
hG01_46
hG01_47
hG01_43
hG01_41
hG01_42
hG01_38
hG01_37
hG01_36
hG01_34
hG01_35
hG01_39
hG01_40
hG01_32
hG01_28
hG01_29
hG01_30
hG01_31
hG01_26
hG01_27
hG01_44
hG01_45
hG01_33
hG01_24
hG01_25
D2 Para. Meso
D2 Card. Meso
D2 Adv. Meso
D3 Mixed. Meso
D3 Para. Meso
D3 Prim. Streak
D3 Early. Meso
D4 Para. Meso
D4 Prim. Streak
D4 Mesenchyme
D4 Somitic Meso
Human gastruloids
Cell types in this study
1
0.8
0.6
0.4
0.2
0
D
Comparison with CS11 non-human primates (32)
Neural Crest
Spinal Cord
Neurons
Cardiomyocytes
Somitic mesoderm−1
Musculoskeletal Progenitors
Somitic mesoderm−2
Urogenital Precursors
Neural Tube
Axial Mesoderm
Endoderm
NC
SC
LP.Meso
Para.Meso
FB.MB.HB
Gut
PS
0.4
0.3
0.2
0.1
0
E
ZIC1
EN2
F
SOX17
FOXC1
TBX6
G
TBXT
MIXL1
EOMES

Supplemental Figure S4: Transcriptional characterization of human gastruloids.

A, Experimental plan showing the transcriptional characterization of human gastruloids (hGs) sampled at different time points. **B,** UMAP of all cell types computed from genes with highly variable expression across all samples represented in Scanorama plot with all annotated clusters at D0, D2, D3, D4 and D8. **C,** Heatmap comparing the transcriptome of D2, D3 and D4 our hGs with previously described human gastruloids (*27*). **D,** Heatmap comparing the transcriptome of Carnegie stage (CS) 11 non-human primate (NHP) embryos to D8 hGs. **E**, UMAP at D0 showing key markers of Ectoderm, including ZIC1 and EN2. **F**, UMAP at D0 showing key markers of anterior and posterior primitive streak (PS), including *SOX17, FOXC1* and *TBX6*. **G,** UMAP at D0 showing key markers of mesendoderm, including *TBXT, MIXL1* and *EOMES*.

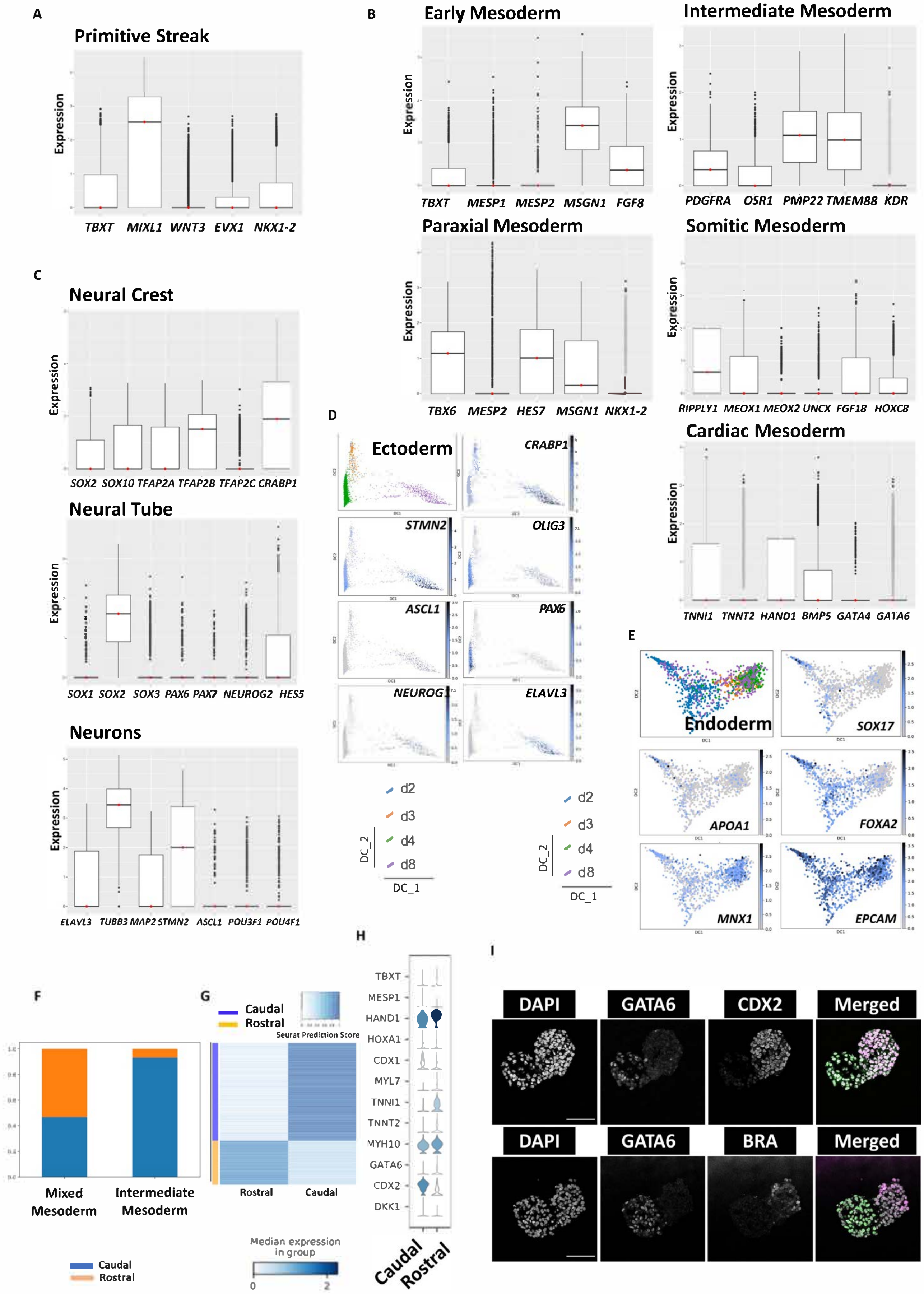
A
Primitive Streak
Expression
TBXT MIXL1 WNT3 EVX1 NKX1-2
B
Early Mesoderm
Expression
TBXT MESP1 MESP2 MSGN1 FGF8
Intermediate Mesoderm
Expression
PDGFRA OSR1 PMP22 TMEM88 KDR
Paraxial Mesoderm
Expression
TBX6 MESP2 HES7 MSGN1 NKX1-2
Somitic Mesoderm
Expression
RIPPLY1 MEOX1 MEOX2 UNCX FGF18 HOXC8
Cardiac Mesoderm
Expression
TNNI1 TNNT2 HAND1 BMP5 GATA4 GATA6
C
Neural Crest
Expression
SOX2 SOX10 TFAP2A TFAP2B TFAP2C CRABP1
Neural Tube
Expression
SOX1 SOX2 SOX3 PAX6 PAX7 NEUROG2 HES5
Neurons
Expression
ELAVL3 TUBB3 MAP2 STMN2 ASCL1 POU3F1 POU4F1
D
Ectoderm
CRABP1
STMN2
OLIG3
ASCL1
PAX6
NEUROG1
ELAVL3
d2
d3
d4
d8
DC_2
DC_1
E
Endoderm
SOX17
APOA1
FOXA2
MNX1
EPCAM
F
Mixed Mesoderm
Intermediate Mesoderm
Caudal
Rostral
G
Caudal
Rostral
Seurat Prediction Score
Rostral
Caudal
Median expression in group
0
2
H
TBXT
MESP1
HAND1
HOXA1
CDX1
MYL7
TNNI1
TNNT2
MYH10
GATA6
CDX2
DKK1
Caudal
Rostral
I
DAPI
GATA6
CDX2
Merged
DAPI
GATA6
BRA
Merged

Supplemental Figure S5: Transcriptional profiles showing the detection of primary germ layer markers in human gastruloids.

A, Box plot showing the detection of key markers of PS, including *TBXT, MIXL1, WNT3, EVX1* and *NKX1-2* in hGs. **B**, Box plots showing the expression of key markers of mesodermal derivatives, including early mesoderm, intermediate mesoderm, paraxial mesoderm, pre-somitic mesoderm and cardiac mesoderm in hGs. **C,** Box plots showing the detection of key markers of ectodermal derivatives, including neural crest, neural tube and neuronal precursors in hGs. **D**, Diffusion component (DC) analysis of Ectoderm derivatives representing key marker expression, including *ASCL1, PAX6, OLIG3, CRABP1, ELAVL3, NEUROG1* and *STMN2*. **E,** Diffusion component (DC) analysis of Endoderm representing key marker expression including *SOX17, FOXA2, MNX1, EPCAM* and *APOA1*. **F**, Fraction of cells showing differential gene expression in mixed mesoderm and intermediate mesoderm of D3 hGs as measured against rostral (anterior) versus caudal (posterior) gene expression in advanced mesoderm cluster of Carnegie stage (CS) 7 human gastrula. **G,** Heatmap showing the comparison of rostral and caudal genes in the advanced mesoderm cluster of CS7 human gastrula versus Intermediate and mixed mesoderm clusters in D3 hGs. **H**, Volcano plots showing the rostral and caudal genes expressed in D3 hGs analysed in (F-G). **I**, hG sections showing an elongation along the anterior-posterior (rostral-caudal) axis represented by the expression of GATA6 and CDX2 (top) and GATA6 and BRA (bottom), respectively. Scale bar, 100µm.

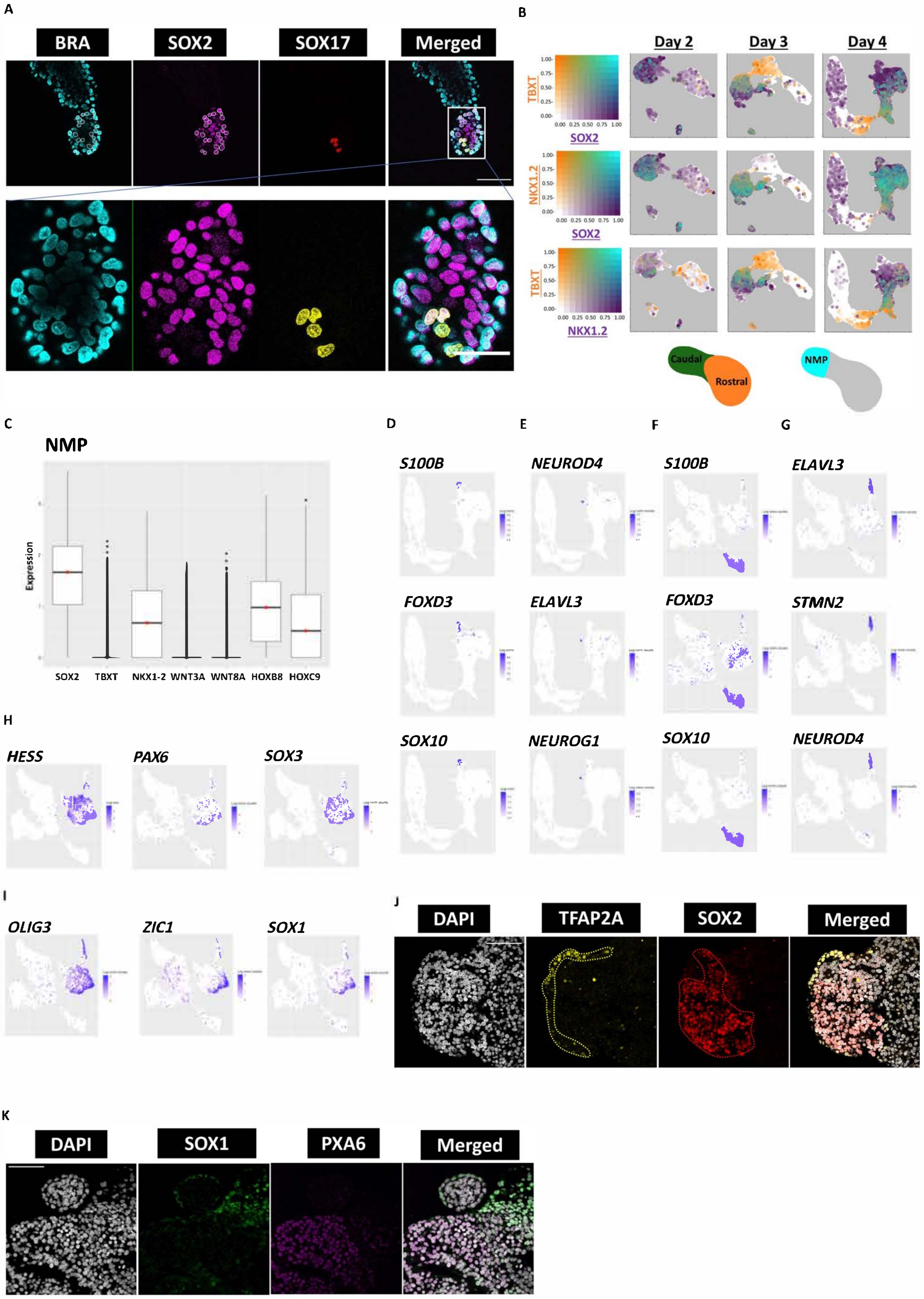
A
BRA
SOX2
SOX17
Merged
B
Day 2
Day 3
Day 4
TBXT
SOX2
NKX1.2
SOX2
TBXT
NKX1.2
Caudal
Rostral
NMP
C
NMP
Expression
SOX2
TBXT
NKX1-2
WNT3A
WNT8A
HOXB8
HOXC9
D
E
F
G
S100B
NEUROD4
S100B
ELAVL3
FOXD3
ELAVL3
FOXD3
STMN2
SOX10
NEUROG1
SOX10
NEUROD4
H
HESS
PAX6
SOX3
I
OLIG3
ZIC1
SOX1
J
DAPI
TFAP2A
SOX2
Merged
K
DAPI
SOX1
PXA6
Merged

Supplementary Figure S6: Human gastruloids display an emergence of neuromesodermal progenitors (NMP).

A, Confocal micrographs of IF staining showing putative neuromesodermal progenitors (NMPs) in D3 hGs. Cells (nuclei) co-expressing SOX2 and BRA (putative NMPs) are circled and amplified. Outlined region is magnified below the corresponding image. Scale bar, 50µm. (N=15 from 3 experiments). **B,** UMAPs of D2, D3 and D4 hGs obtained from 10X scRNAseq dataset showing the co-expression of key NMP markers *TBXT, SOX2 and NKX1-2*. Schematic diagrams showing the potential localization of NMPs in the posterior region of hGs (bottom panel). Color codes indicate (co-)expression profile of the specified genes (left panel). **C**, Box plot showing the expression of key NMP markers detected in scRNAseq dataset, including *SOX2, NKX1.2, TBXT, HOXB8* and *HOXC9*. **D-E,** Confocal micrographs of IF staining showing the emergence of neural precursor markers (SOX1 and PAX6) (D) and neural crest markers (SOX2, TFAP2A) (E) on D8 hG sections, respectively. Scale bar, 100µm (D) 50µm (E). (N=10 from 2 experiments). **F-I**, UMAPs showing the detection of key markers of (F, H) Neural crest and (G, I) neuronal precursors on D4 and D8 hGs datasets, respectively. **J-K,** UMAPs showing the detection of (J) Neural tube markers and (k) spinal cord markers on D8 hGs. In panels F-K, bad quality clusters are also shown.

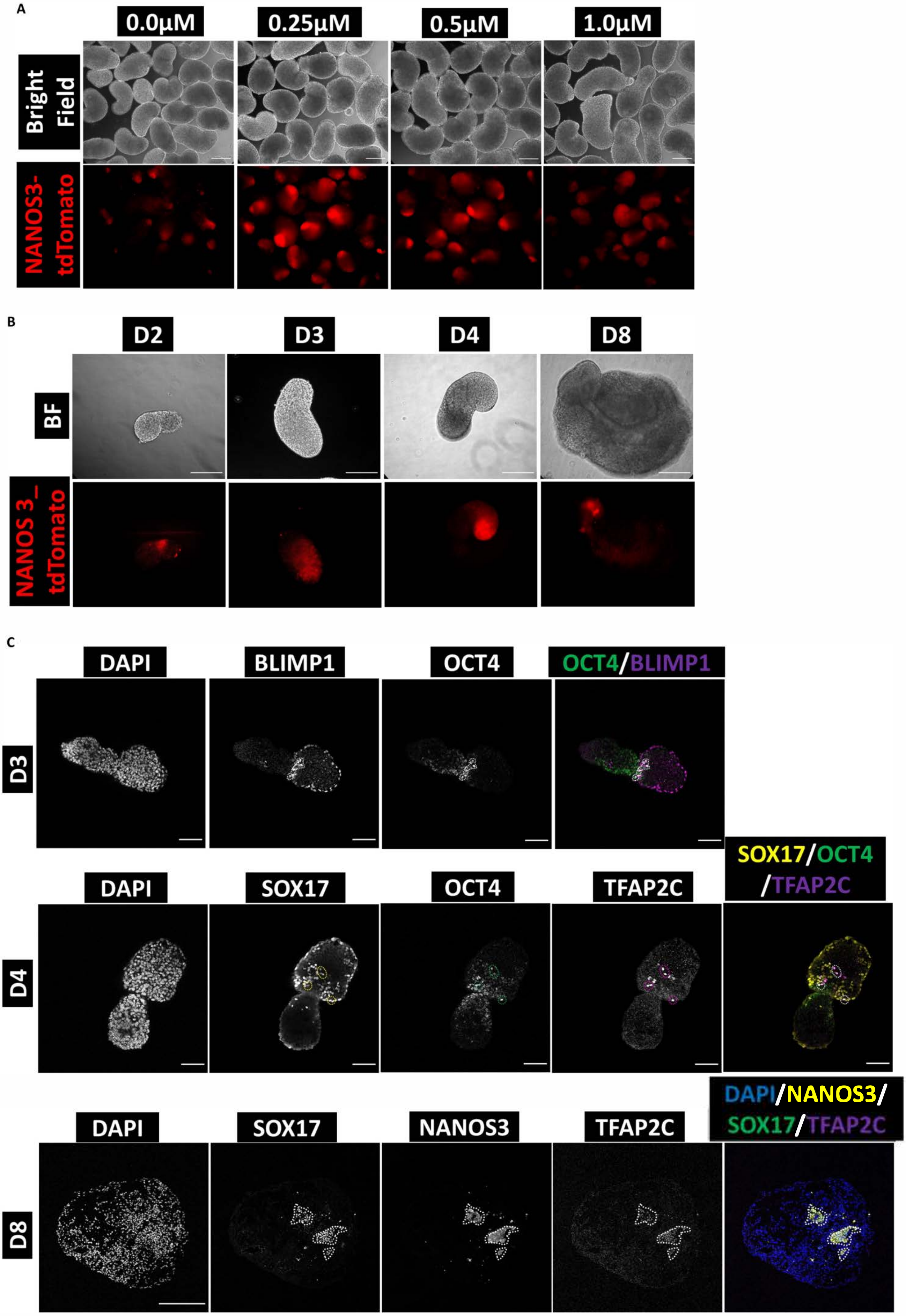
A
0.0µM
0.25µM
0.5µM
1.0µM
Bright Field
NANOS3-tdTomato
B
D2
D3
D4
D8
BF
NANOS 3_ tdTomato
C
DAPI
BLIMP1
OCT4
OCT4/BLIMP1
D3
DAPI
SOX17
OCT4
TFAP2C
SOX17/OCT4 /TFAP2C
D4
DAPI
SOX17
NANOS3
TFAP2C
DAPI/NANOS3/ SOX17/TFAP2C
D8

Supplementary Figure S7: Detection of hPGCLC in the absence of exogenous BMP supplementation in human gastruloids.

A, Microscopic images showing the effect of *Wnt* agonist, *Chiron*, for the specification of hPGCLCs, as shown by the expression of NANOS3 on D3 hGs. Scale bar, 100µm. **B**, Microscopic images showing the detection of hPGCLCs in the absence of exogenous BMP, as shown by NANOS3 reporter in hGs. Scale bar, 100µm. **C**, Confocal micrographs showing the expression of key hPGCLC markers, BLIMP1 (or PRDM1) and POU5F1 (or OCT4); SOX17, OCT4 and BLIMP1 and SOX17, NANOS3 (RFP), TFAP2C in hG sections on D3, D4 and D8, respectively. Scale bar, 100µm.

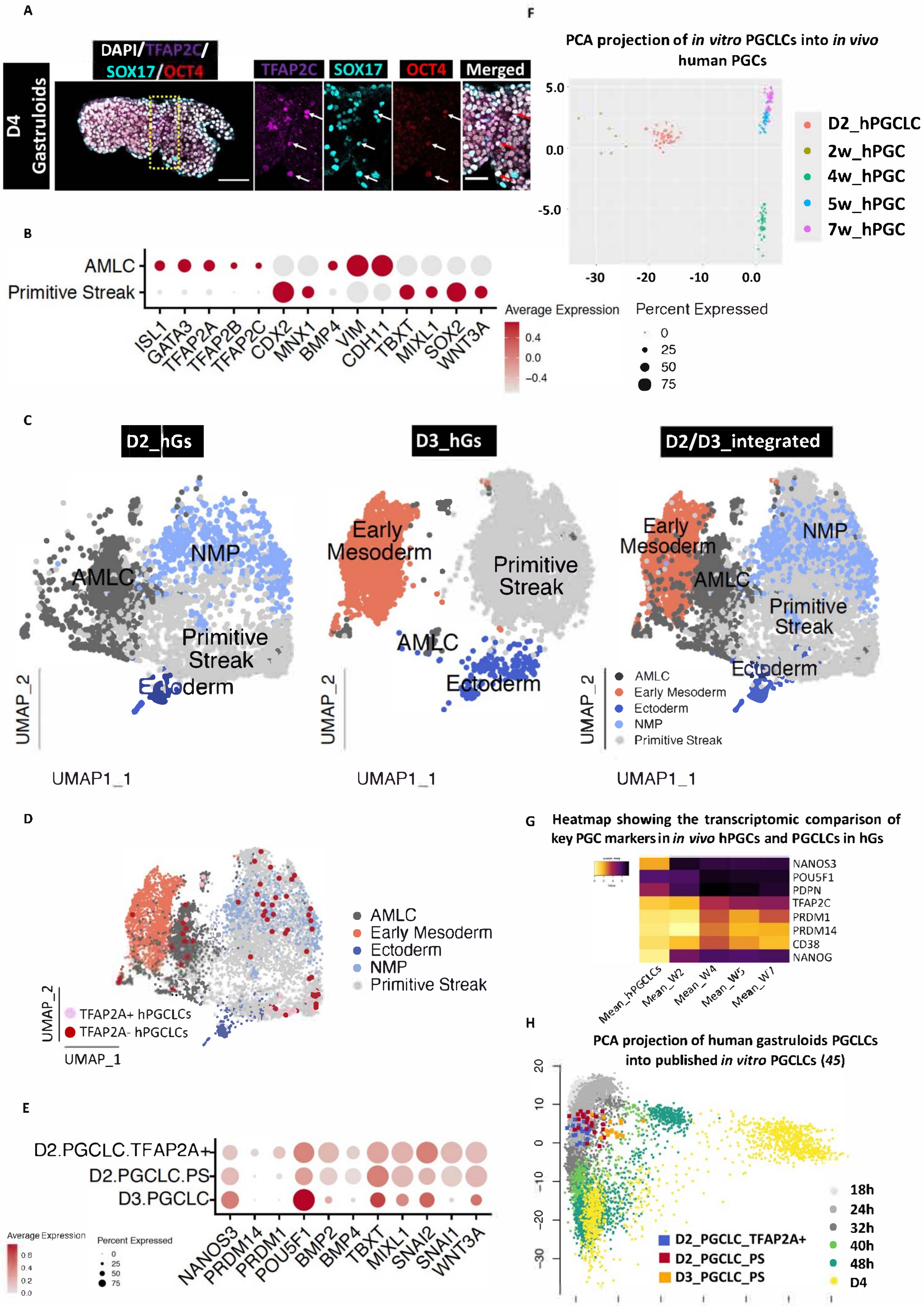

A
DAPI/TFAP2C/SOX17/OCT4
TFAP2C
SOX17
OCT4
Merged
D4 Gastruloids
B
AMLC
Primitive Streak
ISL1
GATA3
TFAP2A
TFAP2B
TFAP2C
CDX2
MNX1
BMP4
VIM
CDH11
TBXT
MIXL1
SOX2
WNT3A
Average Expression
Percent Expressed
C
D2_hGs
D3_hGs
D2/D3_integrated
AMLC
NMP
Primitive Streak
Ectoderm
Early Mesoderm
UMAP_2
UMAP1_1
D
AMLC
Early Mesoderm
Ectoderm
NMP
Primitive Streak
TFAP2A+ hPGCLCs
TFAP2A- hPGCLCs
UMAP_2
UMAP_1
E
D2.PGCLC.TFAP2A+
D2.PGCLC.PS
D3.PGCLC
NANOS3
PRDM14
PRDM1
POU5F1
BMP2
BMP4
TBXT
MIXL1
SNAI2
SNAI1
WNT3A
Average Expression
Percent Expressed
F
PCA projection of in vitro PGCLCs into in vivo human PGCs
D2_hPGCLC
2w_hPGC
4w_hPGC
5w_hPGC
7w_hPGC
G
Heatmap showing the transcriptomic comparison of key PGC markers in in vivo hPGCs and PGCLCs in hGs
NANOS3
POU5F1
PDPN
TFAP2C
PRDM1
PRDM14
CD38
NANOG
Mean_hPGCLCs
Mean_W2
Mean_W4
Mean_W5
Mean_W7
H
PCA projection of human gastruloids PGCLCs into published in vitro PGCLCs (45)
D2_PGCLC_TFAP2A+
D2_PGCLC_PS
D3_PGCLC_PS
18h
24h
32h
40h
48h
D4

Supplementary Figure S8: Detection of hPGCLCs in the absence of exogenous BMP supplementation in human gastruloids.

A, Confocal micrographs showing the expression of key hPGCLC markers TFAP2C, POU5F1 (or OCT4) and SOX17 in D4 hGs. Scale bar, 100µm. **B**, Dot plot showing key amnion-like cells (AMLC) and primitive streak (PS) markers detected in Day 2 and Day 3 hGs identified from scRNAseq dataset. **C**, Sub-clustering and reintegration of primitive streak (PS), early mesoderm (EM), amnion-like cells (AMLCs), ectoderm and neuromesodermal progenitor (NMP) clusters from D2 and D3 scRNA dataset. **D**, UMAP showing the transcriptional localization of hPGCLCs in AMLCs and PS in D2 and D3 hG dataset as detected by 10X scRNAseq. **E**, Dot plot showing key PGCLC markers detected in Day 2 AMLC, Day 2 PS and Day 3 PS identified from 10X scRNAseq dataset. **F**, Principal component analysis (PCA) projection of PGCLCs detected in Day 2 human gastruloids into *in vivo* human PGCs curated from published datasets. Color code shows different stages of *in vivo* human PGCs, and in vitro human PGCLCs from this study. W, *in vivo* human developmental stage in weeks. **G**, Heatmap showing the transcriptomic comparison of *in vivo* hPGC and PGCLCs from D2 human gastruloids (normalized expression levels are log2[TPM+1]). The mean expression is shown for each time point for human PGC and for hPGCLCs from D2. **H**, PCA projection of *in vitro* PGCLCs detected in human gastrulois into *in vitro* hPGCLCs obtained from a published study (*45*).

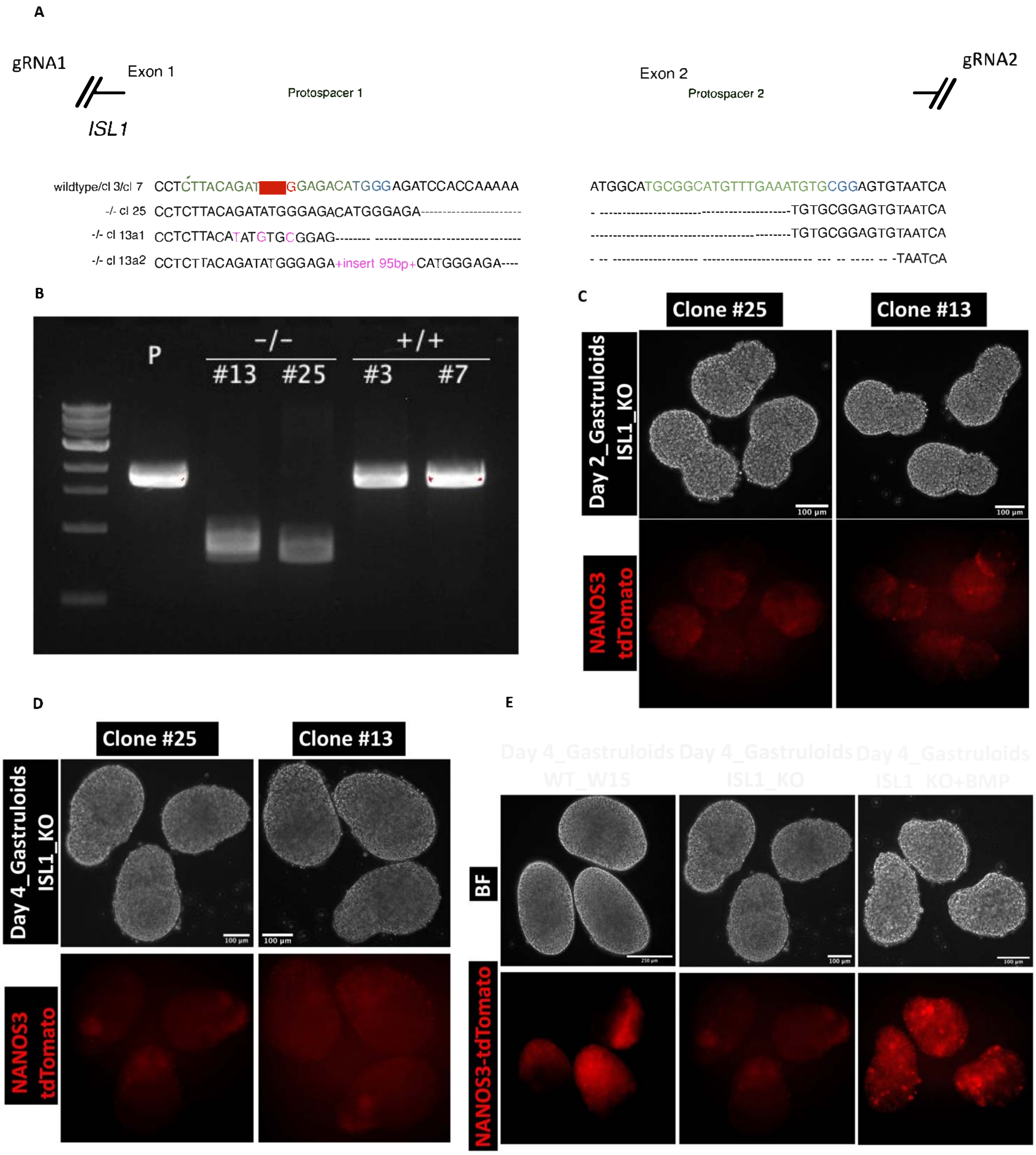
A
gRNA1
Exon 1
ISL1
Protospacer 1
Exon 2
Protospacer 2
gRNA2
wildtype/cl 3/cl 7 CCTCTTACAGAT GGAGACATGGGAGATCCACCAAAAA
-/- cl 25 CCTCTTACAGATATGGGAGACATGGGAGA----------------------
-/- cl 13a1 CCTCTTACATATGTGCGGAG-------- ----------------------------
-/- cl 13a2 CCTCTTACAGATATGGGAGA+insert 95bp+CATGGGAGA----
ATGGCATGCGGCATGTTTGAAATGTGCGGAGTGTAATCA
- ---TGTGCGGAGTGTAATCA
---TGTGCGGAGTGTAATCA
- -- ------------------ ------------------------ --- -- ----- -- -TAATCA
B
P
-/-
#13 #25
+/+
#3 #7
C
Clone #25
Clone #13
Day 2_Gastruloids ISL1_KO
NANOS3 tdTomato
100 µm
D
Clone #25
Clone #13
Day 4_Gastruloids ISL1_KO
NANOS3 tdTomato
100 µm
E
Day 4_Gastruloids WT_W1S
Day 4_Gastruloids ISL1_KO
Day 4_Gastruloids ISL1_KO+BMP
BF
NANOS3-tdTomato
250 µm
100 µm

Supplementary Figure S9: ISL1 knock-out human gastruloids fail to form PGCLC.

A, NCBI sequences showing the generation of ISL1-KO W15-NANOS3-tdTomato hESCs. **B**, Agarose gel electrophoresis image showing the ISL1 KO (#13 and #25) and wildtype (WT) clones (#3 and #7). **C-D**, Live microscopic images showing the lack of fluorescence reporter NANOS3-tdTomato in the ISL1 KO human gastruloids at day 2 and day 4. NANOS3-tdTomato is rescued in gastruloids treated with exogenous BMP supplementation. Scale bar, 100µm. (N=300 samples from 5 experiments). **E**, Live microscopic images showing the detection of fluorescence reporter NANOS3-tdTomato in wildtype but absent in the ISL1 KO human gastruloids at day 4. NANOS3-tdTomato is rescued in gastruloids treated with exogenous BMP supplementation. Scale bar, WT-250µm, KO, KO+BMP-100µm. (N=300 samples from 5 experiments).

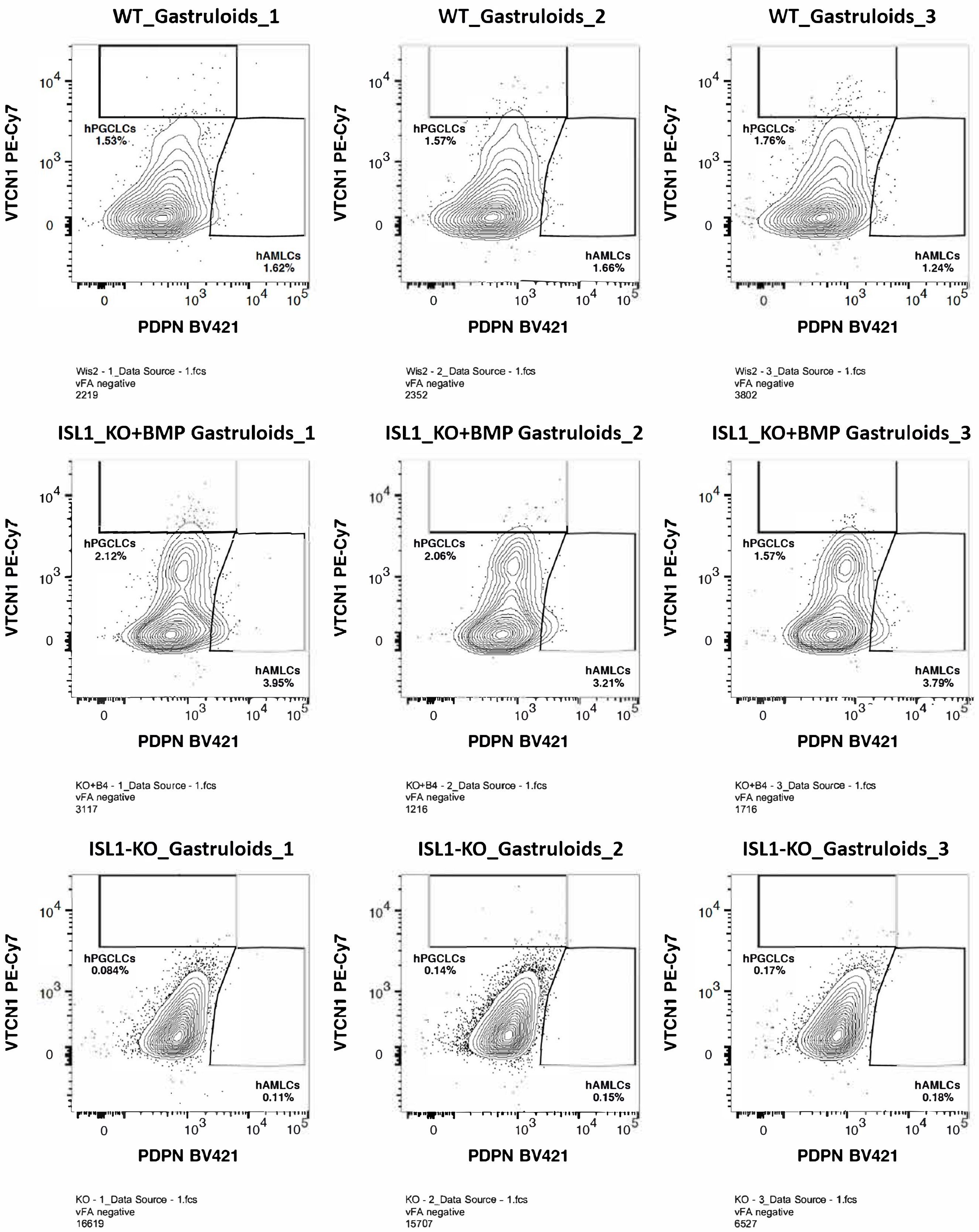
WT_Gastruloids_1
WT_Gastruloids_2
WT_Gastruloids_3
VTCN1 PE-Cy7
PDPN BV421
hPGCLCs 1.53%
hAMLCs 1.62%
hPGCLCs 1.57%
hAMLCs 1.66%
hPGCLCs 1.76%
hAMLCs 1.24%
Wis2 - 1_Data Source - 1.fcs
vFA negative
2219
Wis2 - 2_Data Source - 1.fcs
vFA negative
2352
Wis2 - 3_Data Source - 1.fcs
vFA negative
3802
ISL1_KO+BMP Gastruloids_1
ISL1_KO+BMP Gastruloids_2
ISL1_KO+BMP Gastruloids_3
hPGCLCs 2.12%
hAMLCs 3.95%
hPGCLCs 2.06%
hAMLCs 3.21%
hPGCLCs 1.57%
hAMLCs 3.79%
KO+B4 - 1_Data Source - 1.fcs
vFA negative
3117
KO+B4 - 2_Data Source - 1.fcs
vFA negative
1216
KO+B4 - 3_Data Source - 1.fcs
vFA negative
1716
ISL1-KO_Gastruloids_1
ISL1-KO_Gastruloids_2
ISL1-KO_Gastruloids_3
hPGCLCs 0.084%
hAMLCs 0.11%
hPGCLCs 0.14%
hAMLCs 0.15%
hPGCLCs 0.17%
hAMLCs 0.18%
KO - 1_Data Source - 1.fcs
vFA negative
16619
KO - 2_Data Source - 1.fcs
vFA negative
15707
KO - 3_Data Source - 1.fcs
vFA negative
6527

Supplementary Figure S10: ISL1 knock-out human gastruloids inhibit the formation of PGCLCs and AMLCs.

Flow cytometry analysis panels showing the detection of PGCLCs and AMLCs in the WT, ISL1-KO and KO+BMP supplemented human gastruloids. (N=3 samples from 3 experiments).

A

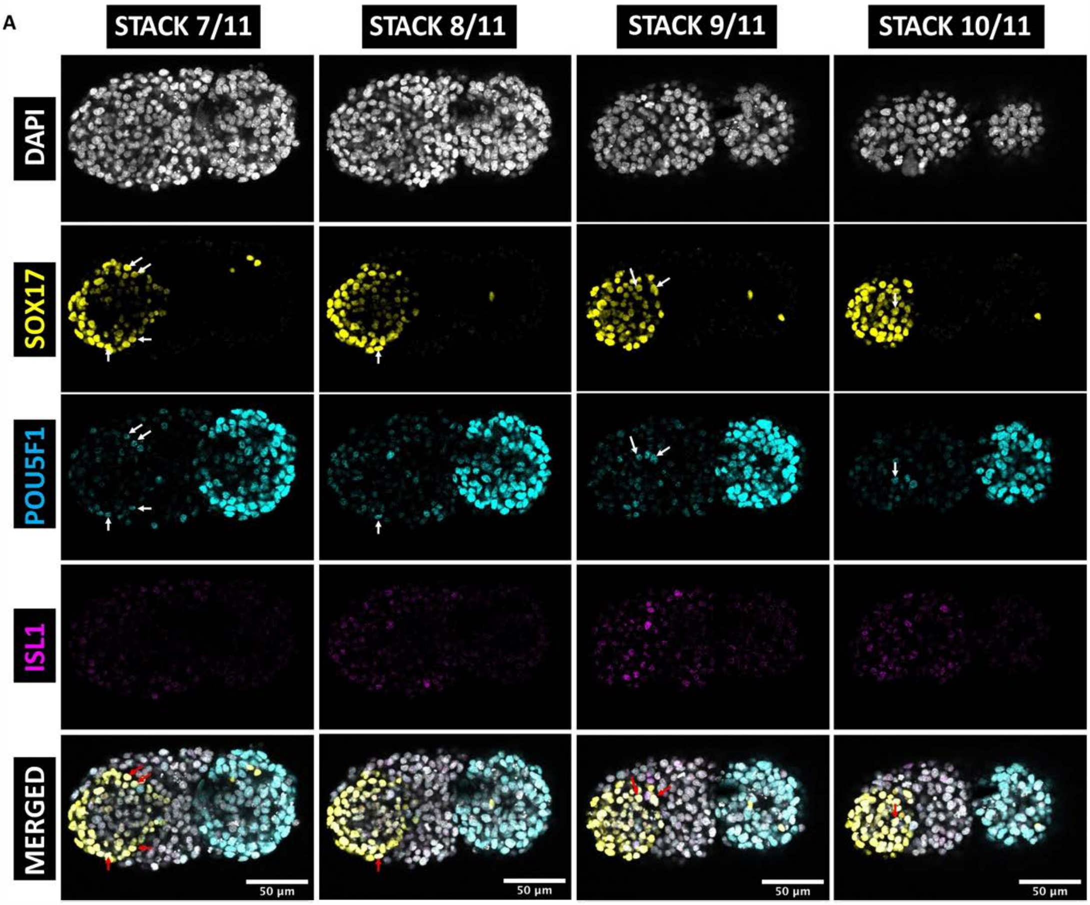
STACK 7/11
STACK 8/11
STACK 9/11
STACK 10/11
DAPI
SOX17
POU5F1
ISL1
MERGED
50 µm
50 µm
50 µm
50 µm

B

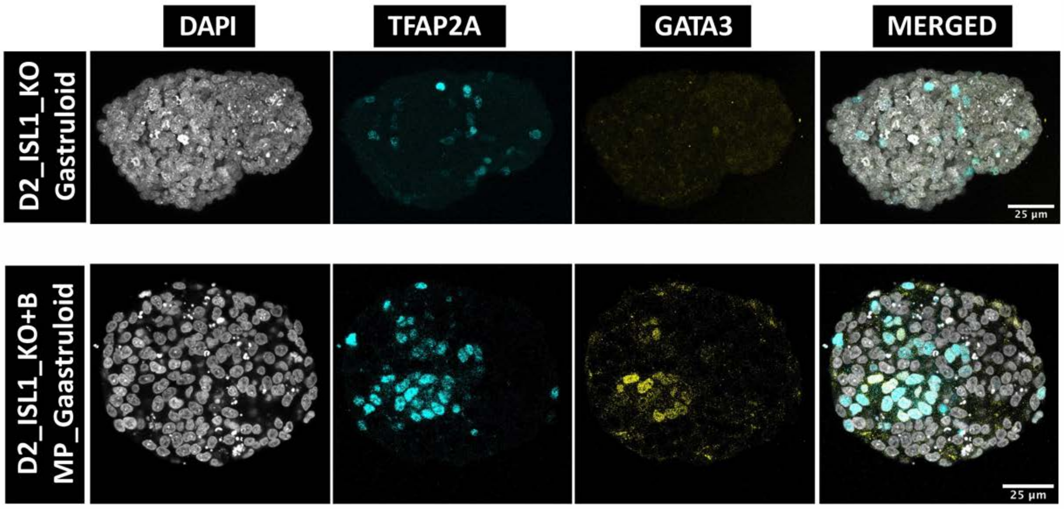
DAPI
TFAP2A
GATA3
MERGED
D2_ISL1_KO Gastruloid
D2_ISL1_KO+BMP_Gaastruloid
25 µm
25 µm

Supplementary Figure S11: Distribution of PGCLCs and AMLCs in human gastruloids

A, Confocal micrographs showing the maximum intensity projection of SOX17, phospho-SMAD 1/5/8 and POU5F1 (OCT4) in the ISL1 KO and KO+BMP supplemented human gastruloids. Scale bar, 50µm. (N=30 samples from 3 experiments). Arrowhead indicates hPGCLCs. **B**, Confocal micrographs showing the maximum intensity projection of TFAP2A and GATA3 in the ISL1 KO and KO+BMP supplemented human gastruloids. Scale bar, 25µm. (N=30 samples from 3 experiments).

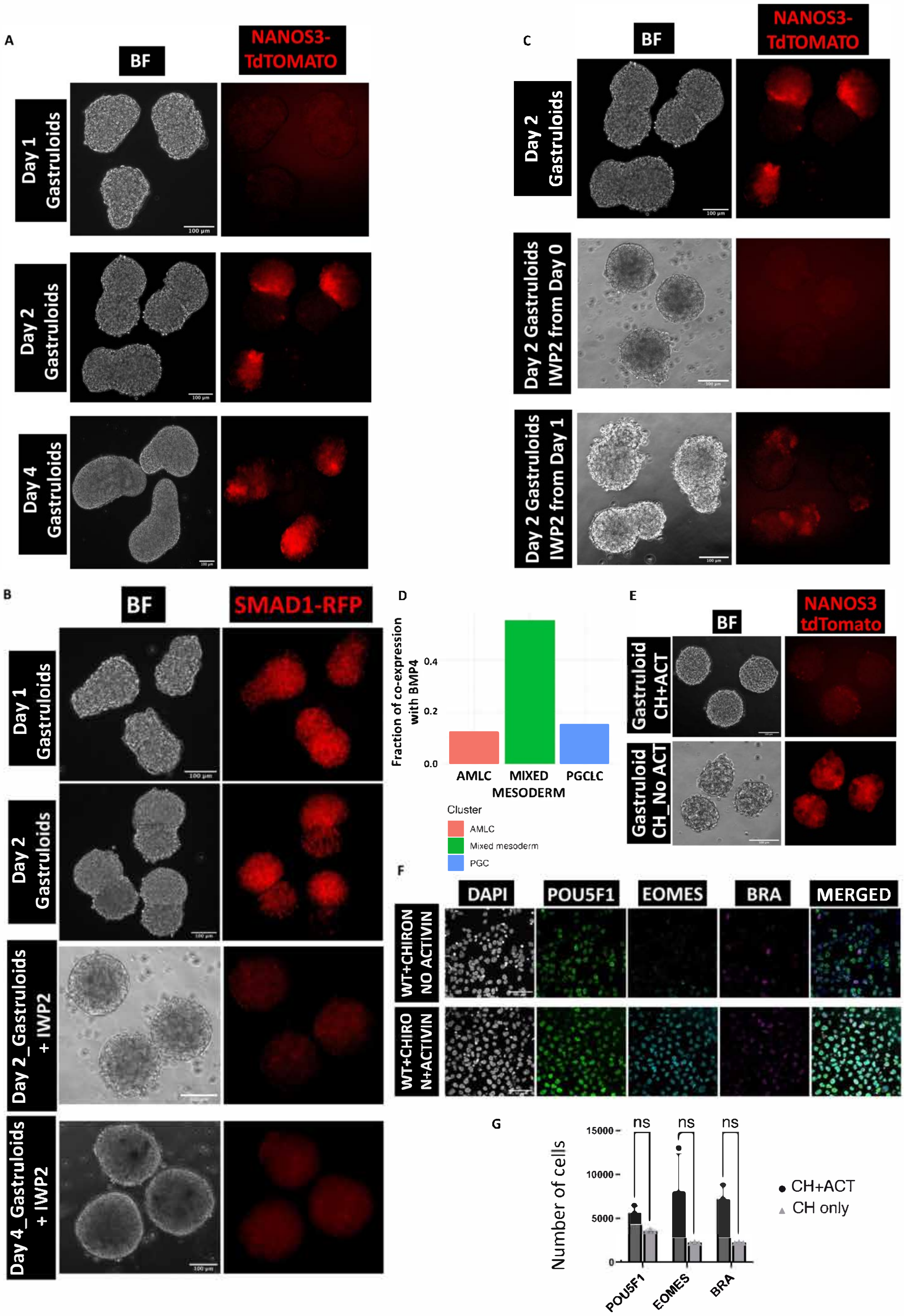
A
BF
NANOS3-TdTOMATO
Day 1 Gastruloids
Day 2 Gastruloids
Day 4 Gastruloids
B
BF
SMAD1-RFP
Day 1 Gastruloids
Day 2 Gastruloids
Day 2_Gastruloids + IWP2
Day 4_Gastruloids + IWP2
C
BF
NANOS3-TdTOMATO
Day 2 Gastruloids
Day 2 Gastruloids IWP2 from Day 0
Day 2 Gastruloids IWP2 from Day 1
D
Fraction of co-expression with BMP4
0.0
0.2
0.4
AMLC
MIXED MESODERM
PGCLC
Cluster
AMLC
Mixed mesoderm
PGC
E
BF
NANOS3 tdTomato
Gastruloid CH+ACT
Gastruloid CH_No ACT
F
DAPI
POU5F1
EOMES
BRA
MERGED
WT+CHIRON NO ACTIVIN
WT+CHIRO N+ACTIVIN
G
Number of cells
15000
10000
5000
0
ns
ns
ns
POU5F1
EOMES
BRA
CH+ACT
CH only

Supplementary Figure S12: WNT and ACTIVIN is required for maintaining germline competency.

A, Live microscopic images showing the detection of NANOS3-tdTomato from day 2 human gastruloids. Scale bar, 100µm. (N=100 samples from 3 experiments). **B**, Live microscopic images showing the inhibition of PGC marker NANOS3-tdTomato in human gastruloids treated with Wnt inhibitor, IWP2. Scale bar, 100µm. (N=100 samples from 3 experiments). **C**, Live microscopic images showing the detection of SMAD1-RFP from day 1 in human gastruloids. Treatment with Wnt inhibitor, IWP2 inhibits the detection of SMAD1-RFP. Scale bar, 100µm. (N=100 samples from 3 experiments). **D**, A bar graph showing the fraction of cells (AMLCs, PGCLCs and mixed mesoderm) co-expressing at least two markers together with BMP4 in day 2 human gastruloids. AMLCs markers: 'GATA3', 'TFAP2A', 'TFAP2C', 'MSX2', 'STOM'; mixed mesoderm markers: 'TBXT', 'PDGFRA', TMEM88', 'MESP1', 'MESP2', 'KDR', 'MSGN1'; PGCLCs markers: 'PDPN', 'NANOS3', 'PRDM1', 'CD38', 'PECAM1'. Color code denotes the cell types. **E**, Live microscopic images showing the detection of NANOS3-tdTomato in human gastruloids generated from hESCs treated with and without Activin for 24h before aggregation. Scale bar, 100µm. (N=60 from 3 experiments). **F**, Immunofluorescence staining showing the presence and absence of PGC competency markers, EOMES and Brachyury (BRA) when treated with or Activin during the first 24h induction of hESCs. Scale bar, 50µm. (N=3 samples (15 images) from 3 experiments). **G**, A bar graph showing the quantification of EOMES and Brachyury positive cells in hESCs treated with and without Activin for 24h before aggregation. (N=15 samples from 3 experiments).

Supplementary Table 1: List of genes used to identify putative PGCLCs in human gastruloids

Combination of markers	Number	Selected for further analysis
TFAP2C.PDPN.POU5F1	895	
PRDM1.PDPN.POU5F1	171	
PRDM1.TFAP2C.POU5F1	94	
PRDM1.TFAP2C.PDPN	87	
PRDM14.PDPN.POU5F1	67	
DND1.PDPN.POU5F1	39	
NANOG.PDPN.POU5F1	34	
PDPN.POU5F1.NANOS3	32	YES
UTF1.PDPN.POU5F1	31	
PRDM14.TFAP2C.PDPN	27	
PRDM14.TFAP2C.POU5F1	27	
PECAM1.PDPN.POU5F1	25	
SOX15.PDPN.POU5F1	20	
CD38.PDPN.POU5F1	16	YES
NANOG.TFAP2C.PDPN	12	
NANOG.TFAP2C.POU5F1	12	
UTF1.TFAP2C.POU5F1	12	
UTF1.TFAP2C.PDPN	11	
DND1.TFAP2C.PDPN	10	
DND1.TFAP2C.POU5F1	9	
PECAM1.TFAP2C.PDPN	9	
PECAM1.TFAP2C.POU5F1	9	
SOX15.TFAP2C.PDPN	9	
SOX15.TFAP2C.POU5F1	8	
CD38.TFAP2C.POU5F1	7	YES
PRDM14.PRDM1.PDPN	7	YES
PRDM14.PRDM1.POU5F1	7	YES
CD38.TFAP2C.PDPN	6	YES
PRDM1.NANOG.PDPN	5	YES
PRDM1.NANOG.POU5F1	5	YES
PRDM14.PRDM1.TFAP2C	5	YES
TFAP2C.POU5F1.NANOS3	5	YES
PRDM1.NANOG.TFAP2C	3	YES
PRDM14.NANOG.PDPN	3	
PRDM14.NANOG.POU5F1	3	
SOX15.PRDM1.POU5F1	3	
SOX15.PRDM1.TFAP2C	3	
TFAP2C.PDPN.NANOS3	3	YES
UTF1.PRDM1.PDPN	3	
UTF1.PRDM1.POU5F1	3	
CD38.PRDM1.PDPN	2	YES
CD38.PRDM1.POU5F1	2	
PECAM1.PRDM14.PDPN	2	

PECAM1.PRDM14.POU5F1	2	YES
PRDM14.NANOG.TFAP2C	2	
PRDM14.PRDM1.NANOG	2	
SOX15.PRDM1.PDPN	2	
SOX17.PDPN.POU5F1	2	
UTF1.PRDM1.TFAP2C	2	
**Total**	**1755**	**61**

Supplementary Table 2: List of antibodies used for IF and flow cytometry

**Antibodies**	**Host**	**Supplier**	**Reference Number**
POU5F1 (OCT4)	Mouse	BD Biosciences	611203
NANOG	Rabbit	Peprotech	500-P236
SOX2	Mouse	Santa Cruz	Sc3465823
SOX2	Goat	Abcam	Ab239218
SOX2	Goat	Santa Cruz	Sc17320
BRACHYURY (BRA)	Rabbit	Abcam	Ab209695
SOX17	Goat	R & D Systems	AF1924
SOX17	Rabbit	Cell Signalling Technology	817785
BLIMP1	Rabbit	Cell Signalling Technology	9115
TFAP2C (AP-2Y)	Rabbit	Abcam	Ab218107
TFAP2A (AP2a)	Mouse	Santa Cruz	Sc12762
EOMES	Sheep	R & D Systems	AF6166
SOX1	Goat	R & D Systems	AF3369
GATA6	Goat	R & D Systems	AF1700
CDX2	Rabbit	Cell Signalling Technology	123065
RFP	Mouse	Rockland/Cambridge Bioscience	200-301-379S
ISL1	Rabbit	Invitrogen (Thermofisher Scientific)	PA5-27789
GATA3	Rabbit	Abcam	ab199428
pSMAD1/5/8	Rabbit	Cell Signalling Technology	9516s
VTCN1_PE-CY7 (flow cytometry)	Mouse anti-human	BioLegend	358105
PDPN_BV421 (flow cytometry)	Mouse anti-human	BD Biosciences	5664565

Supplementary Movie 1: Distribution of multilineage cells in a 3D human gastruloid
